# Identification of miR-20b-5p as an inhibitory regulator in cardiac differentiation via TET2 and DNA hydroxymethylation

**DOI:** 10.1186/s13148-024-01653-7

**Published:** 2024-03-15

**Authors:** Ke-Xin Li, Jia-Ru Li, Sheng-Jia Zuo, Xudong Li, Xian-Tong Chen, Pei-Yi Xiao, Hui-Tao Li, Ling Sun, Tao Qian, Hao-Min Zhang, Dongxing Zhu, Xi-Yong Yu, Guojun Chen, Xue-Yan Jiang

**Affiliations:** 1 Affiliated Qingyuan Hospital, Qingyuan People’s Hospital, Guangzhou Municipal and Guangdong Provincial Key Laboratory of Molecular Target & Clinical Pharmacology, School of Pharmaceutical Science, Guangzhou Medical University, Guangzhou, 511436 China; 2 Department of Cardiology, State Key Laboratory of Organ Failure Research, Guangdong Provincial Key Laboratory of Cardiac Function and Microcirculation, Nanfang Hospital, Southern Medical University, Guangzhou, 510515 China; 3 Peking University Cancer Hospital Yunnan, Yunnan Cancer Hospital, The Third Affiliated Hospital of Kunming Medical University, Kunming, 650118 China; 4 Shenzhen Maternity & Child Healthcare Hospital, Shenzhen, 518028 China; 5 Department of Cardiac Pediatrics, Guangdong Provincial Cardiovascular Institute, Guangdong Provincial People’s Hospital, Southern Medical University, Guangzhou, 510280 China

**Keywords:** MicroRNA-20b-5p, Cardiac differentiation, Tet methylcytosine dioxygenase 2 (TET2), DNA hydroxymethylation

## Abstract

**Background:**

Congenital heart disease (CHD) is a prevalent congenital cardiac malformation, which lacks effective early biological diagnosis and intervention. MicroRNAs, as epigenetic regulators of cardiac development, provide potential biomarkers for the diagnosis and treatment of CHD. However, the mechanisms underlying miRNAs-mediated regulation of cardiac development and CHD malformation remain to be further elucidated. This study aimed to explore the function of microRNA-20b-5p (miR-20b-5p) in cardiac development and CHD pathogenesis.

**Methods and results:**

miRNA expression profiling identified that miR-20b-5p was significantly downregulated during a 12-day cardiac differentiation of human embryonic stem cells (hESCs), whereas it was markedly upregulated in plasma samples of atrial septal defect (ASD) patients. Our results further revealed that miR-20b-5p suppressed hESCs-derived cardiac differentiation by targeting tet methylcytosine dioxygenase 2 (TET2) and 5-hydroxymethylcytosine, leading to a reduction in key cardiac transcription factors including *GATA4*, *NKX2.5*, *TBX5*, *MYH6* and *cTnT*. Additionally, knockdown of TET2 significantly inhibited cardiac differentiation, which could be partially restored by miR-20b-5p inhibition.

**Conclusions:**

Collectively, this study provides compelling evidence that miR-20b-5p functions as an inhibitory regulator in hESCs-derived cardiac differentiation by targeting TET2, highlighting its potential as a biomarker for ASD.

**Supplementary Information:**

The online version contains supplementary material available at 10.1186/s13148-024-01653-7.

## Background

Congenital heart disease (CHD) is a predominant cause of neonatal morbidity and mortality, with an incidence ranging from 6 to 13 cases per 1000 live births. CHD comprises a variety of cardiac malformations, including atrial or ventricular septal defects (ASD or VSD), isolated dysplastic valve anomalies, tetralogy of fallot (TOF) and hypoplastic left heart syndrome (HLHS) [1, 2]. Although CHD is recognized as a multifactorial condition with an unclear etiology, it is acknowledged that genetic and environmental factors play a critical role in its development [3]. For example, ASD is caused by spontaneous genetic mutations in certain genes, including *TBX5*, *NKX2.5*, *GATA4*, *NR2F2*, *ACVR1/ALK2* and *CRELD1* [4, 5]. Despite the high prevalence of CHD, its underlying etiology is still poorly understood, and the early biological diagnostic techniques remain insufficient [5]. Therefore, identifying the key regulators of cardiac development and potential biomarkers of CHD could greatly benefit its diagnosis and management. Growing evidence has demonstrated that epigenetic modifications play crucial role in the etiology of CHD [6–8]. During cardiac development, the expression of cardiac genes is coordinated by a complex transcriptional program involving the interactions between cardiac transcription factors and epigenetic regulators [9]. Mutations in these critical components have been identified as major factors contributing to CHD development. Ongoing fundamental and translational research is yielding crucial insights, including the systematic characterization of non-coding transcriptional regulatory elements involved in heart development [10].

MicroRNAs (miRNAs) are small non-coding RNAs that have been shown to be involved in the regulation of CHD [11, 12]. These miRNAs regulate gene expression through epigenetic mechanisms by promoting mRNA degradation and inhibiting mRNA translation. For instance, miR-26a and miR-22 have been identified as potential regulators of CHD by targeting the PTEN gene, leading to Akt activation and contributing to complex CHD such as TOF and bicuspid aortic valve (BAV) [13–15]. In addition, emerging evidence suggests that miRNAs also involve in regulation of cardiac differentiation [16, 17]. The miR-17–92 cluster, for example, has been shown to be both necessary and sufficient for inducing cardiomyocyte proliferation in postnatal and adult hearts [18]. Besides, microRNA-200c has been identified as a novel repressor of differentiation and maturation in human pluripotent stem cell-derived cardiomyocytes by targeting to GATA binding protein 4 [19, 20]. During the differentiation of hESCs into cardiomyocytes, miR-1, miR-133 and miR-499 were observed to be the most differentially expressed miRNAs compared to undifferentiated hESCs [21]. Nevertheless, the identification of the crucial miRNAs and their targeted genes for cardiac development and CHD malformation remains to be further elucidated.

Previous studies have established a correlation between the DNA methylation status of several cardiac genes and the development of CHD [22, 23]. DNA methylation and hydroxymethylation play an essential role in the epigenetic regulation of gene expression, which occurs at cytosines located within CpG dinucleotides. DNA methylation of CpG islands in gene promoter is primarily responsible for maintaining gene silencing and subsequent loss of gene function, whereas DNA hydroxymethylation plays a reverse role [24]. Furthermore, tet methylcytosine dioxygenase 2 (TET2), an enzyme responsible for catalyzing the conversion of the modified DNA base methylcytosine to 5-hydroxymethylcytosin (5hmC), is critical in the regulation of cardiac differentiation [25]. TET2 has been shown to promote embryonic heart development and plays a primary role in the embryogenesis of vertebrates. Cardiac-specific deletion of TET2 and TET3 in mice results in ventricular non-compaction cardiomyopathy with embryonic lethality [26]. Moreover, single-cell RNA sequencing has revealed a decrease in the number of cardiomyocytes and the suppression of transcriptional patterns within the cardiac tissue due to TET2 and TET3 depletion [27]. These previous findings emphasize the vital role of TET2 as an epigenetic regulator in cardiac formation and differentiation. Thus, it is essential to investigate whether and how TET2 and its related miRNAs regulate cardiac differentiation and development, which would be of great significance for elucidating the regulatory networks of CHD.

In this investigation, we performed RNA sequencing to characterize miRNA expression profiles in cardiac-differentiated hESCs and plasma samples of ASD patients. A total of eight miRNAs, including hsa-miR-20b-5p, were identified to remarkably decrease in 12-day cardiac-differentiated hESCs, but increase in the plasma samples of ASD patients. Our results also illustrated that miR-20b-5p targeted TET2 and 5hmC, leading to the inhibition of key cardiac transcription factors (*GATA4*, *NKX2.5*, *TBX5*, *MYH6* and *cTnT*) during 12-day cardiac differentiation of hESCs. Furthermore, the administration of miR-20b-5p inhibitor effectively rescued the suppression in hESCs-derived cardiac differentiation due to TET2 knockdown. Taken together, this study identifies that miR-20b-5p/TET2 axis plays a crucial role in suppression of cardiac development, providing a prospective biomarker and epigenetic mechanism for ASD.

## Results

### Establishment of hESCs-derived in vitro cardiac differentiation

**Fig. 1:**
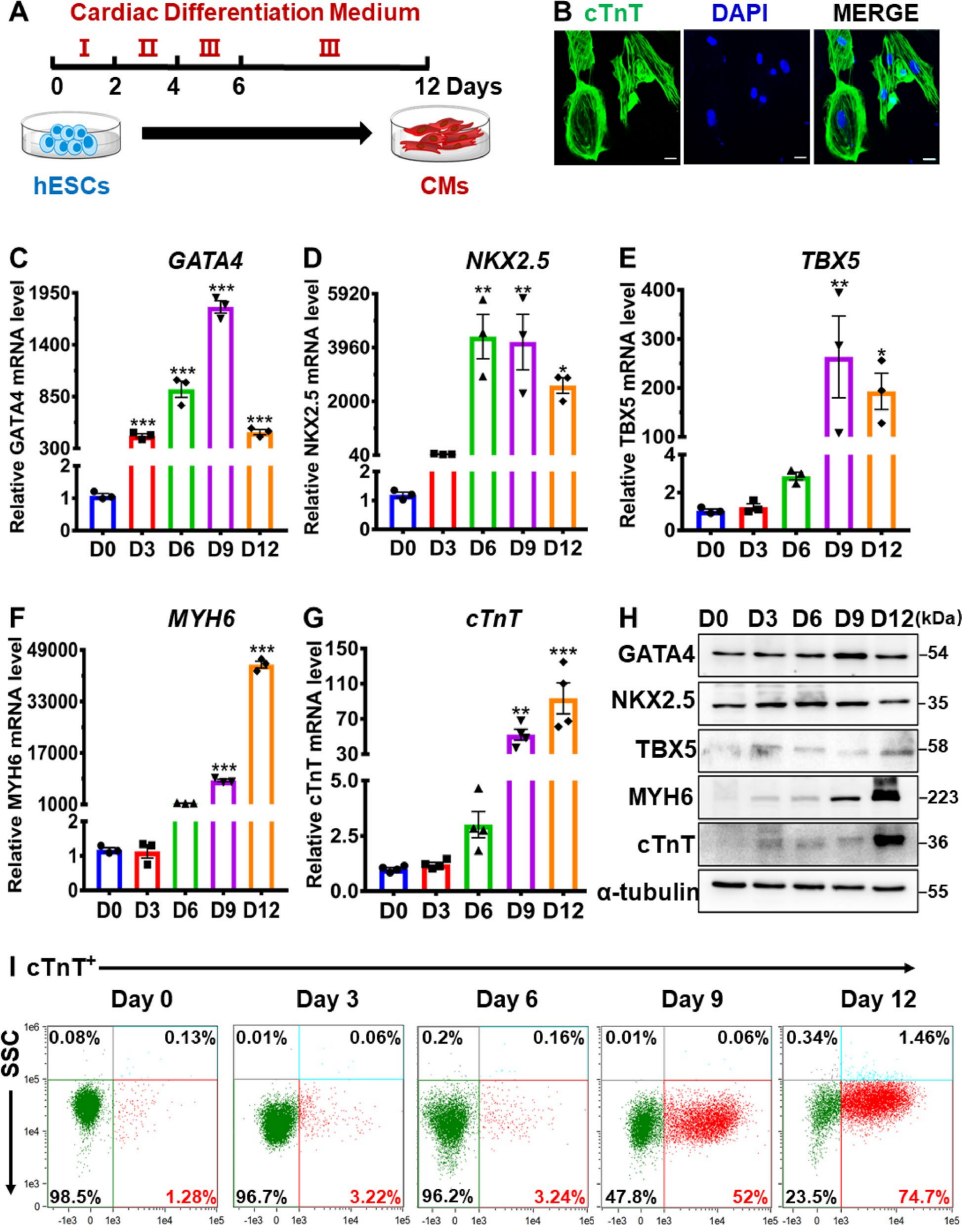
Establishment of hESCs-derived in vitro cardiac differentiation. **A** Scheme of in vitro cardiac differentiation derived from hESCs. **B** Immunofluorescence of cTnT after 12-day differentiation (Scale bar = 20 μm). **C-G** The relative mRNA expression levels of cardiac markers (*GATA4*, *NKX2.5*, *TBX5*, *MYH6* and *cTnT)* during 12-day differentiation were tested by qRT-PCR (*n* = 3-4). **H** The relative protein levels of cardiac markers (GATA4, NKX2.5, TBX5, MYH6 and cTnT) during 12-day differentiation were detected by western blotting (*n* = 3). **I** Flow cytometry analysis of cTnT-positive cell during 12-day differentiation (*n *= 3). D0, D3, D6, D9 and D12 indicated the time points at Day 0, Day 3, Day 6, Day 9 and Day 12, respectively. Quantitative data were presented as mean ± SEM, while statistical significance was analyzed via a one-way ANOVA followed by Bonferroni multiple comparisons test and represented as *P < 0.05, **P < 0.01 and ***P < 0.001

In this study, hESCs-derived cardiomyocytes (hESCs-CMs) were utilized as the primary model for in vitro cardiac differentiation to identify crucial regulators and relative signaling pathways in cardiac development. The scheme of cardiac differentiation and the morphological changes of cells at different time points (Day 0, 3, 6, 9 and 12) during 12-day cardiac differentiation are illustrated in Fig. 1A and Additional file 1: Figure S1. Morphological alterations were observed after treatment with three distinct cardiac differentiation mediums, designated as medium I, II and III. More importantly, the appearance of spontaneously beating cells was observed beginning at day 6 of cardiac differentiation and continued throughout the 12-day duration (Additional file 2: Video 1). To characterize the hESCs-derived cardiac differentiation, the expression of cardiac transcription factors including *GATA4*, *NKX2.5*, *TBX5*, *MYH6* and *cTnT*, which are known to be key regulators in cardiac development, was analyzed [28, 29]. Immunofluorescence showed robust expression of cTnT in hESCs after 12-day cardiac differentiation (Fig. 1B). Moreover, qRT-PCR and western blot analysis demonstrated that the expression of GATA4, NKX2.5, TBX5, MYH6 and cTnT was significantly increased in hESCs during 12-day cardiac differentiation (Fig. 1C to H). The expression levels of early-stage cardiac markers (GATA4, NKX2.5 and TBX5) peaked at day 9 (Fig. 1C to E), while the peak of late-stage cardiac markers (MYH6 and cTnT) emerged at day 12 (Fig. 1F to G). Flow cytometry analysis further revealed that the proportion of cTnT-positive cells increased in a time-dependent manner during the 12-day cardiac differentiation process (Fig. 1I). These findings suggest the successful establishment of a robust in vitro cardiac differentiation system using hESCs, enabling further investigation of the underlying molecular mechanisms of cardiac development.

### MiRNAs expression profiling during hESCs-derived cardiac differentiation

**Fig. 2:**
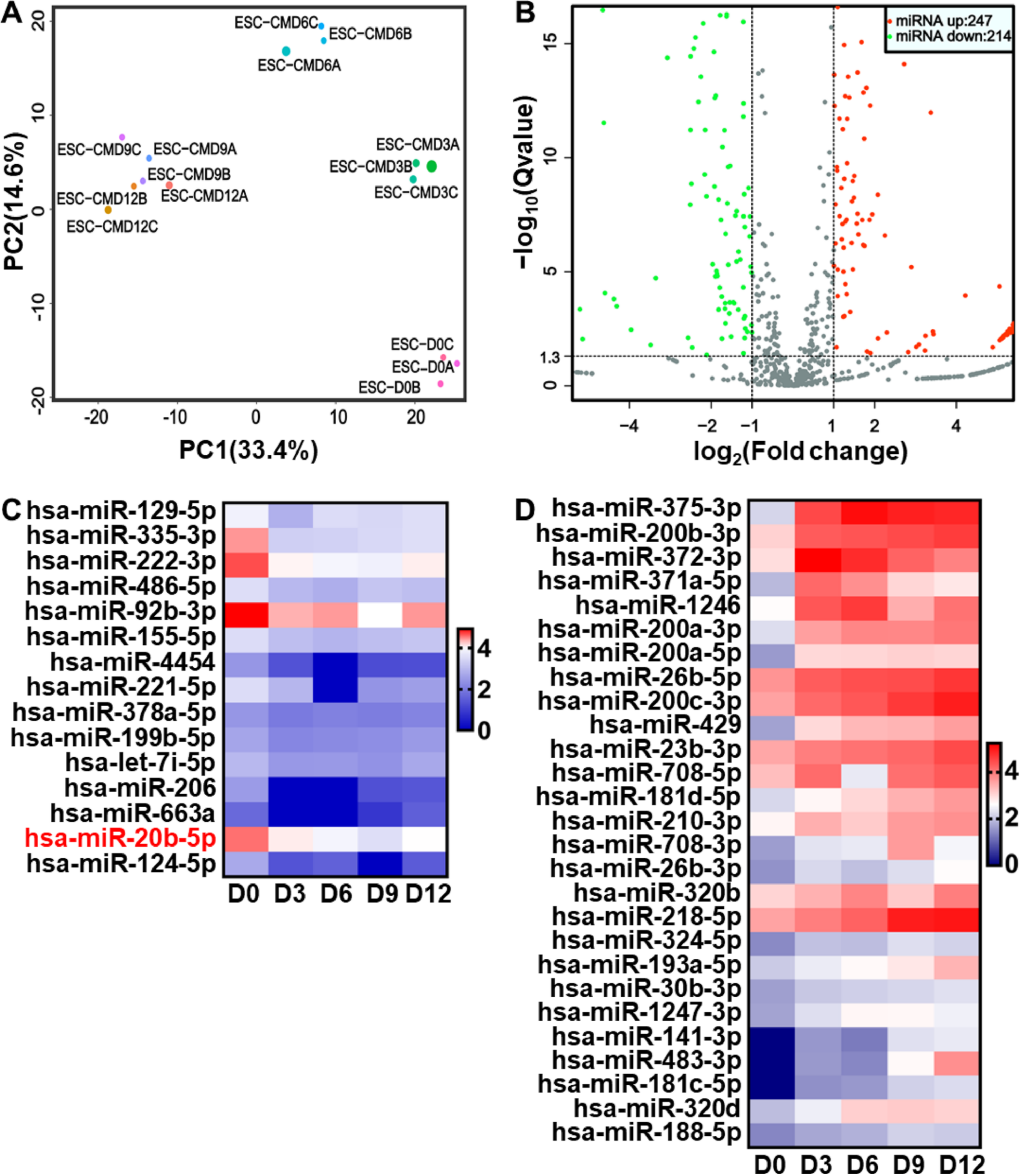
MiRNAs expression profiling during hESCs-derived cardiac differentiation. **A** Principal component (PC) analysis of miRNAs during 12-day cardiac differentiation of hESCs (*n* = 3). **B** Volcano plot of differentially expressed miRNAs in 12-day cardiac-differentiated hESCs. The red and green dots represented significantly upregulated and downregulated miRNAs in Day 12 group, respectively (*n* = 3). **C** Heatmap of remarkably downregulated miRNAs relative to cardiovascular disease during 12-day cardiac differentiation of hESCs (|log2FC|≥ 1.2, *P* < 0.05, *n* = 3). **D** Heatmap of markedly upregulated miRNAs relative to cardiovascular disease during 12-day cardiac differentiation of hESCs (|log2FC|≥ 1.2, *P* < 0.05, *n* = 3). D0, D3, D6, D9 and D12 indicated the time points at Day 0, Day 3, Day 6, Day 9 and Day 12, respectively

To identify differentially expressed miRNAs during cardiac differentiation, we conducted Illumina deep RNA sequencing of cardiac-differentiated hESCs samples on Day 0, 3, 6, 9 and 12. Principal component analysis (PCA) of the RNA-sequencing data revealed that three independent biological repeats of each sample were grouped together, while samples from different groups displayed clear separation (Fig. 2A). A total of 247 miRNAs were significantly upregulated, while 214 miRNAs were remarkably downregulated after 12-day cardiac differentiation of hESCs (|log2 FC|≥ 1, *P* < 0.05) (Fig. 2B). In addition, we identified 46 downregulated miRNAs as well as 48 upregulated miRNAs at each time point of cardiac differentiation including Day 0, 3, 6, 9 and 12 (|log2 FC|≥ 1.2, *P* < 0.05) (Additional file 1: Figures S2A and S3A). KEGG pathway analysis of the downregulated miRNAs illustrated that they were enriched in pathways related to cardiac development, including regulating pluripotency of stem cells and Hippo signaling pathway (Additional file 1: Figure S2B). Furthermore, gene ontology (GO) analysis of these downregulated miRNAs showed enrichment in multicellular organism development, positive regulation of cellular process and other functions (Additional file 1: Figure S2C). Similarly, KEGG pathway analysis of the upregulated miRNAs revealed enrichment in signaling pathways related to cardiac development, such as regulating pluripotency of stem cells, Wnt signaling pathway and Hippo signaling pathway (Additional file 1: Figure S3B). The GO analysis identified that these upregulated miRNAs were enriched in system development and other functions (Additional file 1: Figure S3C). Notably, among those miRNAs downregulated at each time point of hESCs-derived cardiac differentiation, a set of 15 miRNAs including hsa-miR-20b-5p were found to be associated with the regulation of cardiovascular diseases (Fig. 2C and Additional file 1: Table S1). Similarly, 27 miRNAs were screened out of the upregulated miRNAs in 12-day cardiac-differentiated hESCs and were identified to be involved in cardiovascular diseases (Fig. 2D and Additional file 1: Table S2).

### Upregulation of TET2 during in vitro and in vivo cardiac differentiation

**Fig. 3:**
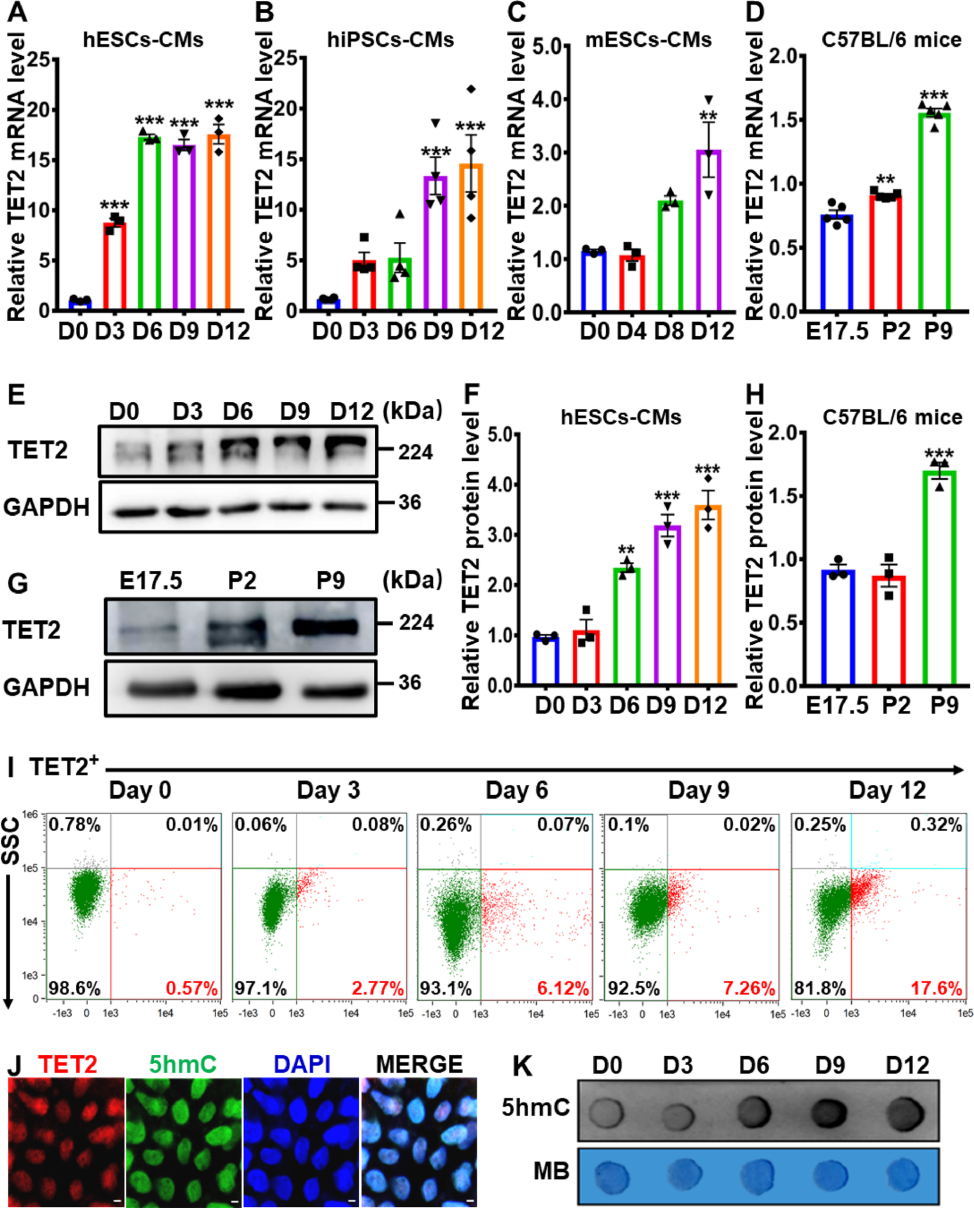
Upregulation of TET2 during in vitro and in vivo cardiac differentiation*.* **A-C** qRT-PCR analysis of the relative *TET2* mRNA level during 12-day cardiac differentiation in hESCs-CMs **A**, hiPSCs-CMs **B** and mESCs-CMs **C** (*n* = 3–4). **D** qRT-PCR analysis of the relative *TET2* mRNA level in embryonic and neonatal heart samples (E17.5, P2 and P9) from wild-type C57BL/6 mice (*n* = 5). **E **and** F** Western blot analysis **E** and quantification **F** of the relative TET2 protein level during 12-day cardiac differentiation of hESCs (*n* = 3). **G **and** H** Western blot analysis **G** and quantification **H** of the relative TET2 protein level in embryonic and neonatal heart samples (E17.5, P2 and P9) from wild-type C57BL/6 mice (*n* = 3). **I** Flow cytometry analysis of TET2-positive cells during 12-day cardiac differentiation of hESCs (*n* = 3). **J** Immunofluorescence of TET2 and 5hmc in 12-day cardiac-differentiated hESCs (Scale bar = 10 μm). **K** Dot blot analysis of global 5hmC level during 12-day cardiac differentiation of hESCs. Methylene blue (MB) staining demonstrated equal loading. D0, D3, D6, D9 and D12 indicated the time points at Day 0, Day 3, Day 6, Day 9 and Day 12, while E17.5, P2 and P9 indicated Embryonic day 17.5, Postnatal day 2 and 9, respectively. Quantitative data were presented as mean ± SEM, while statistical significance was analyzed via a one-way ANOVA followed by Bonferroni multiple comparisons test and represented as *P < 0.05, **P < 0.01 and ***P < 0.001

Previous studies have demonstrated the predominant role of Tet methylcytosine dioxygenase 2 (TET2) in embryonic development. Specifically, TET2 has been found to promote embryonic heart development via regulation of gene expression and chromatin region opening, whereas impaired TET2 function during embryogenesis is associated with fetal death and cardiovascular disease [26, 27, 30]. To investigate the impact of TET2 on cardiac differentiation, the expression of TET2 was evaluated in various in vitro and in vivo cardiac differentiation models. Both mRNA and protein expression levels of TET2 were found to be elevated in several in vitro cardiac differentiation models including human embryonic stem cell-derived cardiomyocytes (hESCs-CMs), human-induced pluripotent stem cell-derived cardiomyocytes (hiPSCs-CMs) and mouse embryonic stem cell-derived cardiomyocytes (mESCs-CMs), as determined by qRT-PCR and western blot analyses (Fig. 3A to C, E and F). Similarly, a significant upregulation of TET2 mRNA and protein expression levels has also been identified during in vivo cardiac development in embryonic and neonatal heart samples from wild-type C57BL/6 mice (Fig. 3D, G and H). Furthermore, flow cytometry analysis showed a time-dependent increase in the ratio of TET2-positive cells during 12-day cardiac differentiation of hESCs (Fig. 3I). Immunofluorescence analysis of TET2 and 5hmC distribution revealed that TET2 was co-localized with 5hmC in the nucleus of 12-day cardiac-differentiated hESCs (Fig. 3J). Additionally, dot blot analysis confirmed the expression of 5hmC increased in a time-dependent manner during 12-day cardiac differentiation of hESCs, which was consistent with TET2 expression level (Fig. 3K). These findings imply that TET2 and its associated DNA hydroxymethylation facilitate cardiac differentiation. As a result, our subsequent studies focus specifically on the role of miRNAs in regulation of TET2 and DNA hydroxymethylation during hESCs-derived cardiac differentiation.

### Identification of miR-20b-5p in plasma samples from ASD patients and 12-day cardiac-differentiated hESCs

**Fig. 4:**
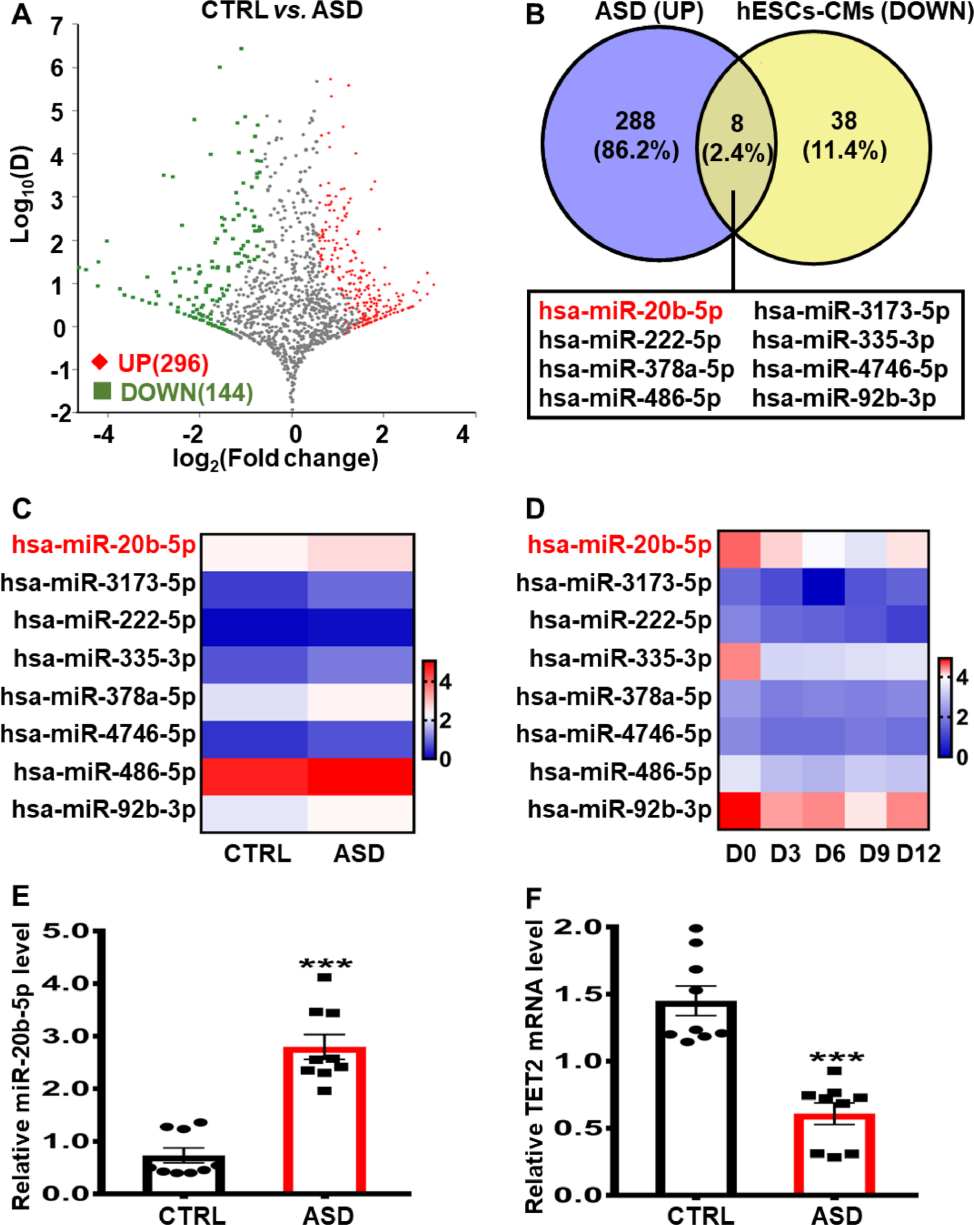
Identification of differentially expressed miR-20b-5p in plasma samples from ASD patients and 12-day cardiac-differentiated hESCs. **A** Volcano plot of differentially expressed miRNAs in plasma samples from ASD patients or healthy controls (*n* = 9–10). The red and green dots represented significantly upregulated and downregulated miRNAs in ASD group, respectively. **B** Schematic illustration of candidate miRNAs significantly upregulated in plasma samples from ASD patients but downregulated in 12-day cardiac-differentiated hESCs. **C** Heatmap of the eight candidate miRNAs upregulated in plasma samples from ASD patients (*n* = 9-10). **D** Heatmap of the eight candidate miRNAs downregulated during 12-day cardiac differentiation of hESCs (*n* = 3). **E** qRT-PCR analysis of miR-20b-5p expression level in plasma samples from ASD patients or healthy controls (*n* = 9). **F** qRT-PCR analysis of the relative *TET2* mRNA expression level in plasma samples from ASD patients or healthy controls (*n* = 9). D0, D3, D6, D9 and D12 indicated the time points at Day 0, Day 3, Day 6, Day 9 and Day 12, respectively. Quantitative data were presented as mean ± SEM, while statistical significance was analyzed via a two-tailed unpaired Student’s t test and represented as *P < 0.05 and ***P < 0.001

Atrial septal defect (ASD) is classified as the third most prevalent type of congenital heart disease [31]. To elucidate the potential involvement of epigenetic regulation of non-coding RNA in the pathogenesis of ASD, we conducted a comprehensive analysis of miRNAs expression using genome-wide RNA sequencing of ribosomal RNAs extracted from plasma samples of ASD patients and healthy controls. Our results revealed 296 significantly upregulated and 144 remarkably downregulated miRNAs in plasma samples from ASD patients compared to healthy controls (Fig. 4A). To identify key miRNAs involved in ASD, we compared the upregulated miRNAs in plasma samples from ASD patients with those miRNAs downregulated in cardiac differentiation of hESCs, leading to the identification of eight candidate miRNAs, including hsa-miR-20b-5p, hsa-miR-3173-5p, hsa-miR-222-5p, hsa-miR-335-3p, hsa-miR-378a-5p, hsa-miR-4746-5p, hsa-miR-486-5p and hsa-miR-92b-3p (Fig. 4B). The differential expression of these eight candidate miRNAs in plasma samples of healthy control or ASD patients, as well as in 12-day cardiac-differentiated hESCs, were shown in Fig. 4C and D, Additional file 1: Tables S3 and S4. To clarify the potential impact of these candidate miRNAs on cardiac differentiation, the interaction between the target regions of the eight candidate miRNAs and TET2 was predicted using TargetScan. Of these candidates, the predicted binding site of miR-20b-5p to TET2 mRNA was the most reliable, with a context score percentile ranking of 86. Also, the probability of preferentially conserved targeting (P_CT_) indicated that miR-20b-5p was the most conserved among the candidate miRNAs (Additional file 1: Table S5). The qRT-PCR analysis further confirmed that miR-20b-5p was significantly upregulated, while the mRNA level of TET2 was remarkably reduced in plasma samples of ASD patients compared to healthy controls (Fig. 4E and F). These results suggest that miR-20b-5p may play a critical role in regulating cardiac differentiation via targeting TET2.

### Inhibition of hESCs-derived cardiac differentiation by miR-20b-5p

**Fig. 5:**
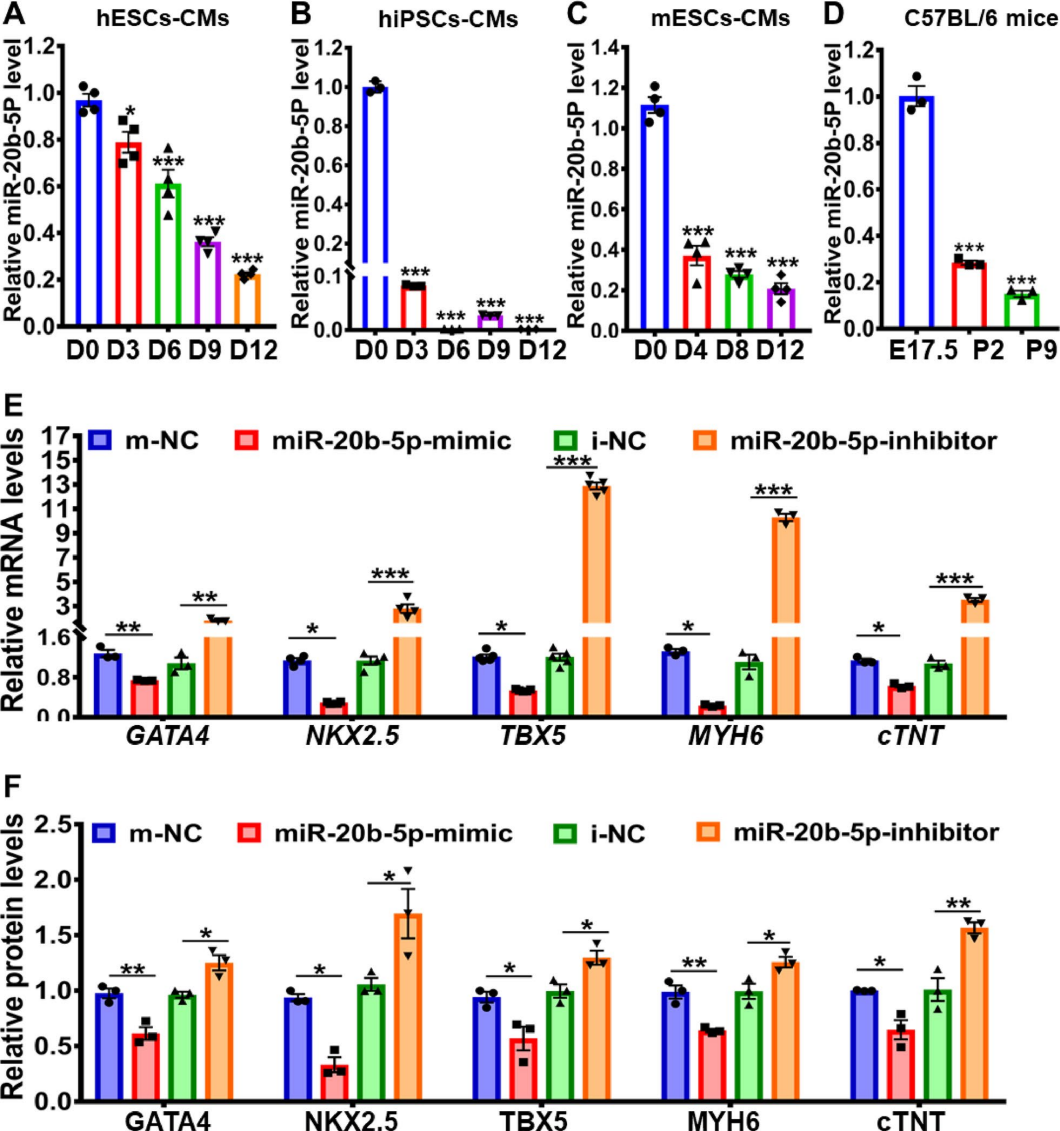

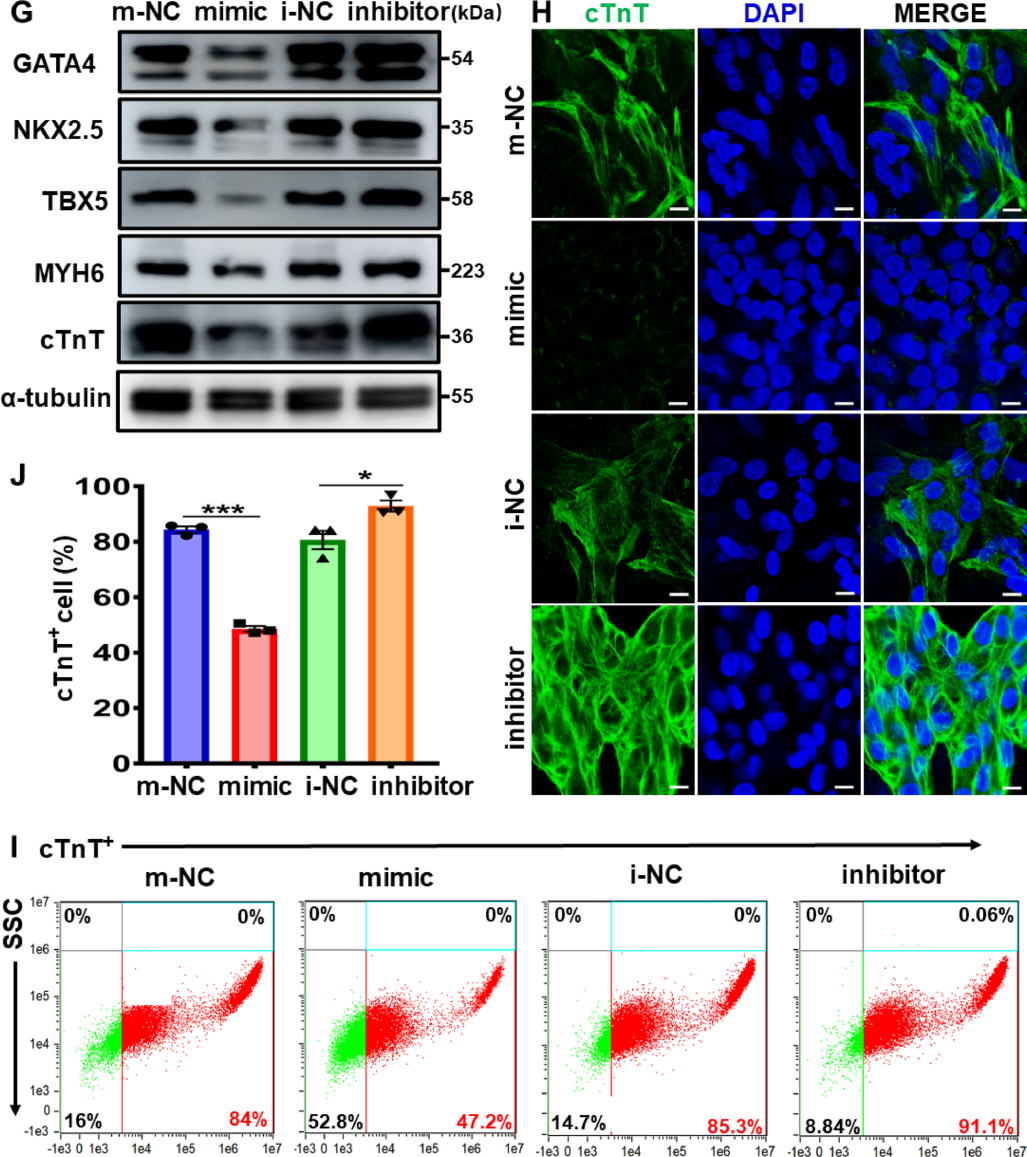
Inhibition of hESC-derived cardiac differentiation by miR-20b-5p. **A-C** qRT-PCR analysis of miR-20b-5p level during 12-day cardiac differentiation in hESCs-CMs **A**, hiPSCs-CMs **B** and mESCs-CMs **C** (*n* = 3–4). **D** qRT-PCR analysis of miR-20b-5p level in embryonic and neonatal heart samples (E17.5, P2 and P9) from wild-type C57BL/6 mice (*n* = 3). **E** qRT-PCR analysis of the relative mRNA expression level of cardiac transcriptional factors (*GATA4*, *NKX2.5*, *TBX5*, *MYH6* and *cTnT*) in 12-day cardiac-differentiated hESCs with treatment of m-NC (mimic-negative control), miR-20b-5p-mimic, i-NC (inhibitor-negative control) or miR-20b-5p-inhibitor (*n* = 3–5). **F** and** G** Western blot analysis **G** and quantification **F** of the relative protein levels of cardiac transcriptional factors (GATA4, NKX2.5, TBX5, MYH6 and cTnT) in 12-day cardiac-differentiated hESCs with treatment of m-NC, miR-20b-5p-mimic, i-NC or miR-20b-5p-inhibitor (*n* = 3). **H** Immunofluorescence of cTnT in 12-day cardiac-differentiated hESCs with treatment of m-NC, miR-20b-5p-mimic, i-NC or miR-20b-5p-inhibitor (Scale bar = 10 μm). **I** and** J** Flow cytometry analysis **I** and quantification **J** of cTnT-positive cells in 12-day cardiac-differentiated hESCs with treatment of m-NC, miR-20b-5p-mimic, i-NC or miR-20b-5p-inhibitor (*n* = 3). D0, D3, D6, D9 and D12 indicated the time points at Day 0, Day 3, Day 6, Day 9 and Day 12, while E17.5, P2 and P9 indicated Embryonic day 17.5, Postnatal day 2 and 9, respectively. Quantitative data were presented as mean ± SEM, while statistical significance was analyzed via a one-way ANOVA followed by Bonferroni multiple comparisons test and represented as *P < 0.05, **P < 0.01 and ***P < 0.001

Although the upregulation of miR-20b-5p in plasma samples from ASD patients and its downregulation in 12-day cardiac-differentiated hESCs have been identified by miRNA expression profiling, the effect of miR-20b-5p on the hESCs-derived cardiac differentiation and the regulatory pathway have not yet been fully elucidated. To provide further insights into the role of miR-20b-5p in cardiac differentiation, this study employed several in vitro and in vivo cardiac differentiation models, including 12-day cardiac differentiation in hESCs-CMs, hiPSCs-CMs and mESCs-CMs. Given the highly conserved sequence of miR-20b-5p in both human and mouse genomes (Additional file 1: Table S6), in vivo cardiac development models of embryonic and neonatal hearts from wild-type C57BL/6 mice were also used to assess the role of miR-20b-5p in heart development. The qRT-PCR analysis revealed a time-dependent decrease in miR-20b-5p during in vitro 12-day cardiac differentiation of hESCs-CMs, hiPSCs-CMs and mESCs-CMs (Fig. 5A to C), which was consistent with the results of miRNA profiling. Similarly, a time-dependent reduction of miR-20b-5p was observed in the in vivo cardiac development of embryonic and neonatal hearts (E17.5, P2 and P9) from wild-type C57BL/6 mice (Fig. 5D).

To further confirm the regulation of miR-20b-5p in hESCs-derived cardiac differentiation, this study conducted overexpression and knockdown experiments of miR-20b-5p in hESCs-CMs. Firstly, the optimal transfection concentration for miR-20b-5p mimic and inhibitor was determined as 100 nM and 300 nM, respectively, after testing a serial of transfection conditions (Additional file 1: Figure S4A and B). The transfection efficiency of miR-20b-5p mimic on hESCs-CMs was also confirmed by transfection at 100 nM every 2 days during 12-day cardiac differentiation (Additional file 1: Figure S4C). Subsequently, the effects of miR-20b-5p on the expression of key cardiac transcription factors (GATA4, NKX2.5, TBX5, MYH6 and cTnT) in hESCs-derived cardiac differentiation were investigated. The results showed that the administration of miR-20b-5p mimic significantly reduced the mRNA and protein levels of these cardiac transcription factors in 12-day cardiac-differentiated hESCs, indicating that miR-20b-5p inhibited hESCs-derived cardiac differentiation (Fig. 5E to G). Conversely, the administration of miR-20b-5p inhibitor positively regulated the expression of these cardiac transcription factors. Immunofluorescence analysis further demonstrated that the miR-20b-5p mimic decreased the expression of cTnT in 12-day cardiac-differentiated hESCs compared to the negative control, while the treatment with miR-20b-5p inhibitor restored the positive expression of cTnT (Fig. 5H). Flow cytometry analysis revealed that the treatment with miR-20b-5p mimic led to a significant decrease of 36.8% in the proportion of cTnT-positive cells, while the administration of miR-20b-5p inhibitor resulted in a notable increase of 5.8% in the ratio of cTnT-positive cells (Fig. 5I to J). Taken together, these findings suggest that miR-20b-5p acts as a major suppressive regulator during hESCs-derived cardiac differentiation by inhibiting the expression of key cardiac transcription factors.

### MiR-20b-5p-mediated reduction of TET2 and 5hmC in cardiac-differentiated hESCs via targeting TET2

**Fig. 6:**
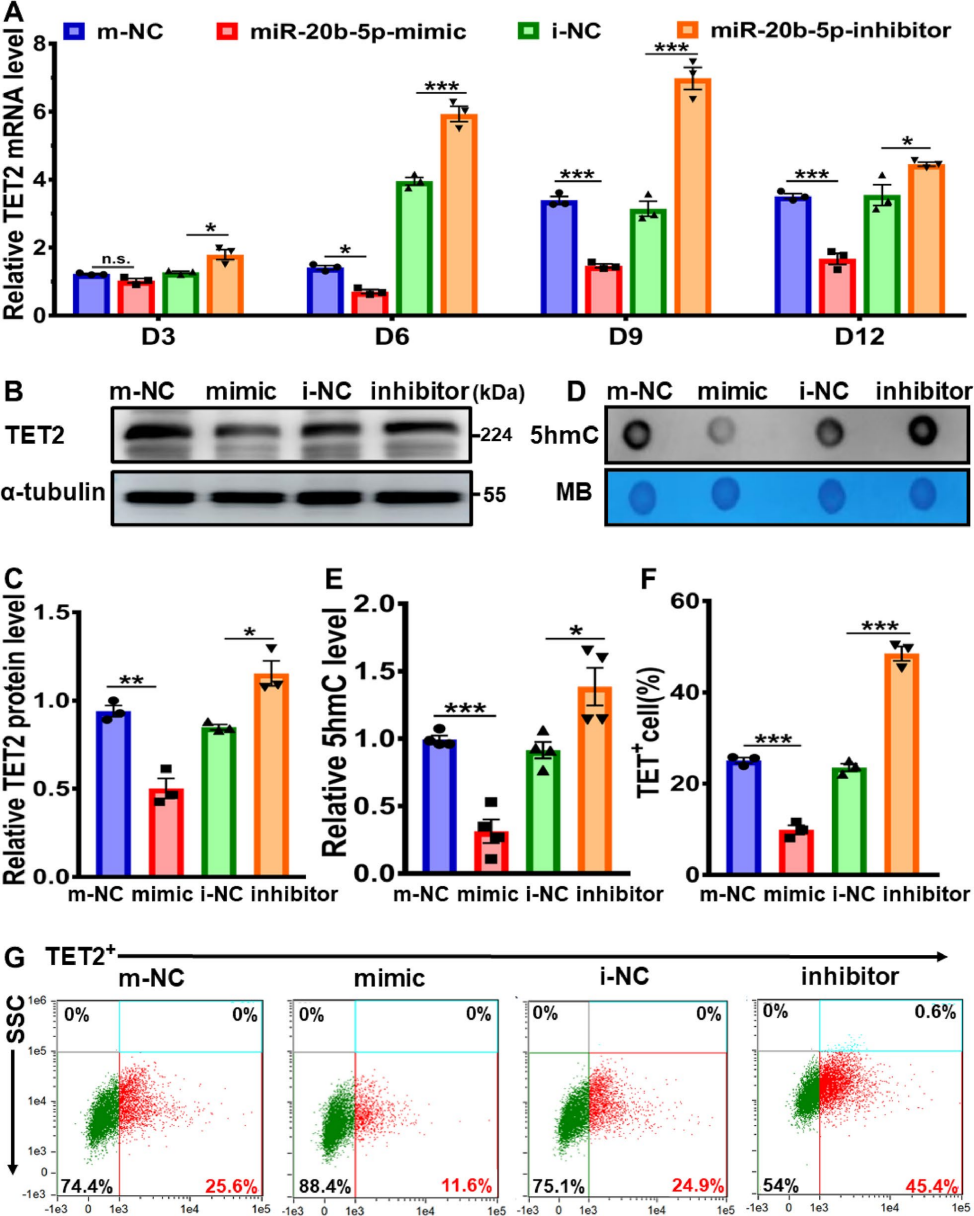

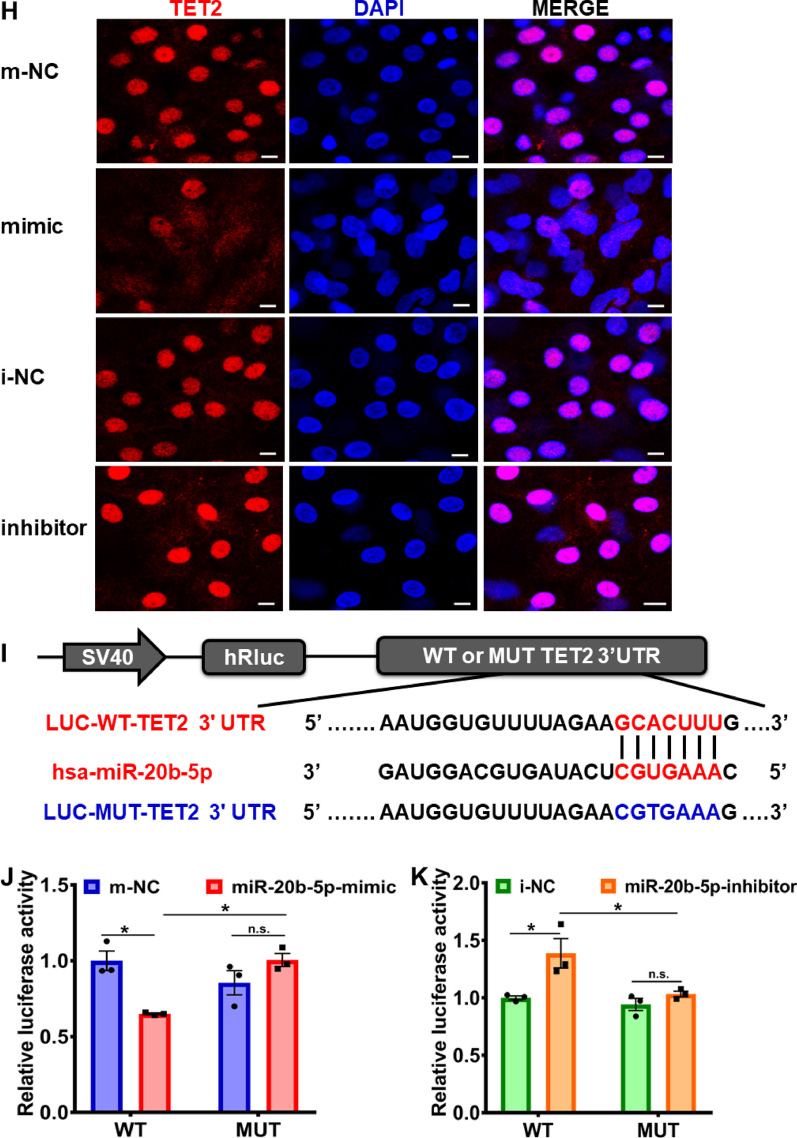
Direct targeting of TET2 by miR-20b-5p during cardiac differentiation of hESCs. **A** qRT-PCR analysis of the relative *TET2* mRNA expression level during 12-day cardiac differentiation of hESCs with exposure to m-NC (mimic-negative control), miR-20b-5p-mimic, i-NC (inhibitor-negative control) or miR-20b-5p-inhibitor (*n* = 3). **B** and** C** Western blot analysis **B** and quantification **C** of the relative TET2 protein level in 12-day cardiac-differentiated hESCs with exposure to m-NC, miR-20b-5p-mimic, i-NC or miR-20b-5p-inhibitor (*n* = 3). **D **and** E** Dot blot analysis **D** and quantification **E** of 5hmC expression in 12-day cardiac-differentiated hESCs with exposure to m-NC, miR-20b-5p-mimic, i-NC or miR-20b-5p-inhibitor (*n* = 4). Methylene blue staining demonstrated equal loading. **F** and** G** Flow cytometry analysis **G** and quantification **F** of TET2-positive cells in 12-day cardiac-differentiated hESCs with exposure to m-NC, miR-20b-5p-mimic, i-NC or miR-20b-5p-inhibitor (*n* = 3). **H** Immunofluorescence staining of TET2 in 12-day cardiac-differentiated hESCs with exposure to m-NC, miR-20b-5p-mimic, i-NC or miR-20b-5p-inhibitor (Scale bar = 10 μm). **I** The dual luciferase reporter system was utilized to construct the predicted binding sequences of miR-20-5p to the 3’ untranslated region of TET2 (LUC-WT-TET2) and a corresponding mutated sequence (LUC-MUT-TET2). **J** and** K** The relative luciferase activity was detected in HEK-293 T cells transfected with LUC-WT-TET2 or LUC-MUT-TET2 with exposure to m-NC, miR-20b-5p-mimic, i-NC or miR-20b-5p inhibitor (*n* = 3). D3, D6, D9 and D12 indicated the time points at Day 3, Day 6, Day 9 and Day 12, respectively. Quantitative data were presented as mean ± SEM. Statistical significance was analyzed via a one-way ANOVA followed by Bonferroni multiple comparisons test and represented as *P < 0.05, **P < 0.01 and ***P < 0.001, while n.s. indicated non-significance

The post-transcriptional regulation of mRNA expression by miRNAs is well established, whereby miRNAs bind to a specific sequence within the 3' untranslated region (3'UTR), known as the seed sites, and then inhibit mRNA expression [32]. In order to examine the hypothesis that TET2 is a direct target of miR-20b-5p, as predicted by TargetScan (Additional file 1: Table S5), we treated 12-day cardiac-differentiated hESCs with miR-20b-5p mimic and inhibitor and analyzed the expression of TET2. As depicted in Fig. 6A to C, the administration of miR-20b-5p mimic significantly reduced both mRNA and protein expression levels of TET2 during 12-day differentiation of hESCs at different time points (Day 3, 6, 9 and 12), while the treatment of miR-20b-5p inhibitor exerted an enhancing effect on TET2 expression. Notably, the global 5hmC level was diminished in 12-day cardiac-differentiated hESCs following treatment with miR-20b-5p mimic, but significantly increased after exposure to miR-20b-5p inhibitor (Fig. 6D and E). Additionally, flow cytometry and immunofluorescence analyses were utilized to confirm the inhibitory effect of miR-20b-5p mimic and the enhancing effect of miR-20b-5p inhibitor on TET2 expression (Fig. 6F to H). The percentage of TET2-positive cell population significantly decreased by 14.0% with the treatment of miR-20b-5p mimic, while remarkably increased by 20.5% with exposure to miR-20b-5p inhibitor (Fig. 6F and G). In line with this, a decline of TET2 fluorescence intensity was detected in 12-day cardiac-differentiated hESCs treated with miR-20b-5p mimic, whereas a slight increase in fluorescence intensity was observed following exposure to miR-20b-5p inhibitor using immunofluorescence (Fig. 6H).

In our previous investigation, it was revealed that TargetScan predicted the potential targeting of the TET2 gene by miR-20b-5p (Additional file 1: Table S5). To further study the potential binding sites of miR-20b-5p in the 3'UTR of TET2 mRNA, a dual luciferase reporter assay was conducted. Specifically, a 524 bp sequence of TET2 (NM_001127208.3, 3’UTR: 413–936) was inserted into a pmiR-RB-ReportTM luciferase reporter and designated as LUC-WT-TET2, while a corresponding mutated sequence without predicted miR-20b-5p binding sites was constructed and named LUC-MUT-TET2 (Fig. 6I). As demonstrated in Fig. 6J and K, the treatment of miR-20b-5p mimic significantly reduced the luciferase activity in LUC-WT-TET2 group, whereas the miR-20b-5p inhibitor exposure increased the luciferase activity. In contrast, no effect on the luciferase activity was observed in LUC-MUT-TET2 group after transfection with miR-20b-5p mimic or inhibitor (Fig. 6J and K). These findings support the notion that miR-20b-5p acts as a suppressor of TET2, resulting in the decrease in TET2 and 5hmC in cardiac differentiation of hESCs.

### Reversal of TET2 knockdown-induced suppression in hESCs-derived cardiac differentiation by inhibiting miR-20b-5p

In order to further explore the impact of miR-20b-5p on TET2-regulated cardiac differentiation, a lentivirus vector (LV) expressing shRNA of TET2 (LV-TET2-shRNA) and enhanced green fluorescent protein (eGFP) was employed to knockdown TET2 in hESCs (Additional file 1: Figure S5A). The efficiency of TET2 knockdown was confirmed by the significant decrease in both TET2 mRNA and protein expression levels in the LV-shTET2 group compared to the negative control of the lentivirus vector (LV-NC) group, indicating the effectiveness of TET2 knockdown in hESCs (Additional file 1: Figure S5B and S5C). The downregulation of TET2 was also observed during the 12-day cardiac differentiation at specific time points (Day 6, 9 and 12) in the LV-shTET2 group, further confirming the successful knockdown of TET2 (Additional file 1: Figure S5D). To clarify the effect of miR-20b-5p on cardiac differentiation in TET2 knockdown hESCs, the cells were subsequently subjected to a 12-day cardiac differentiation, during which either a miR-20b-5p mimic or inhibitor was transfected.

**Fig. 7:**
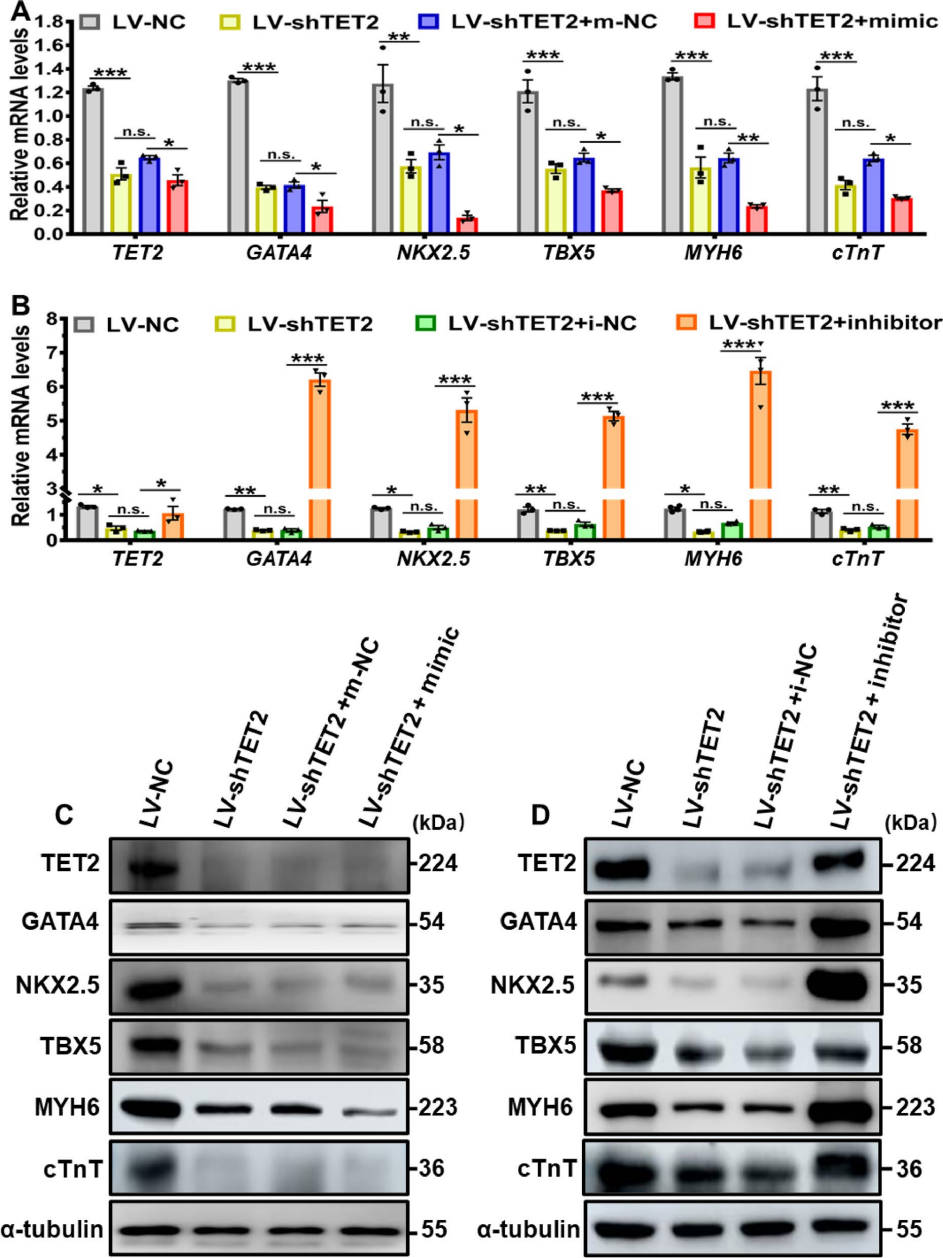

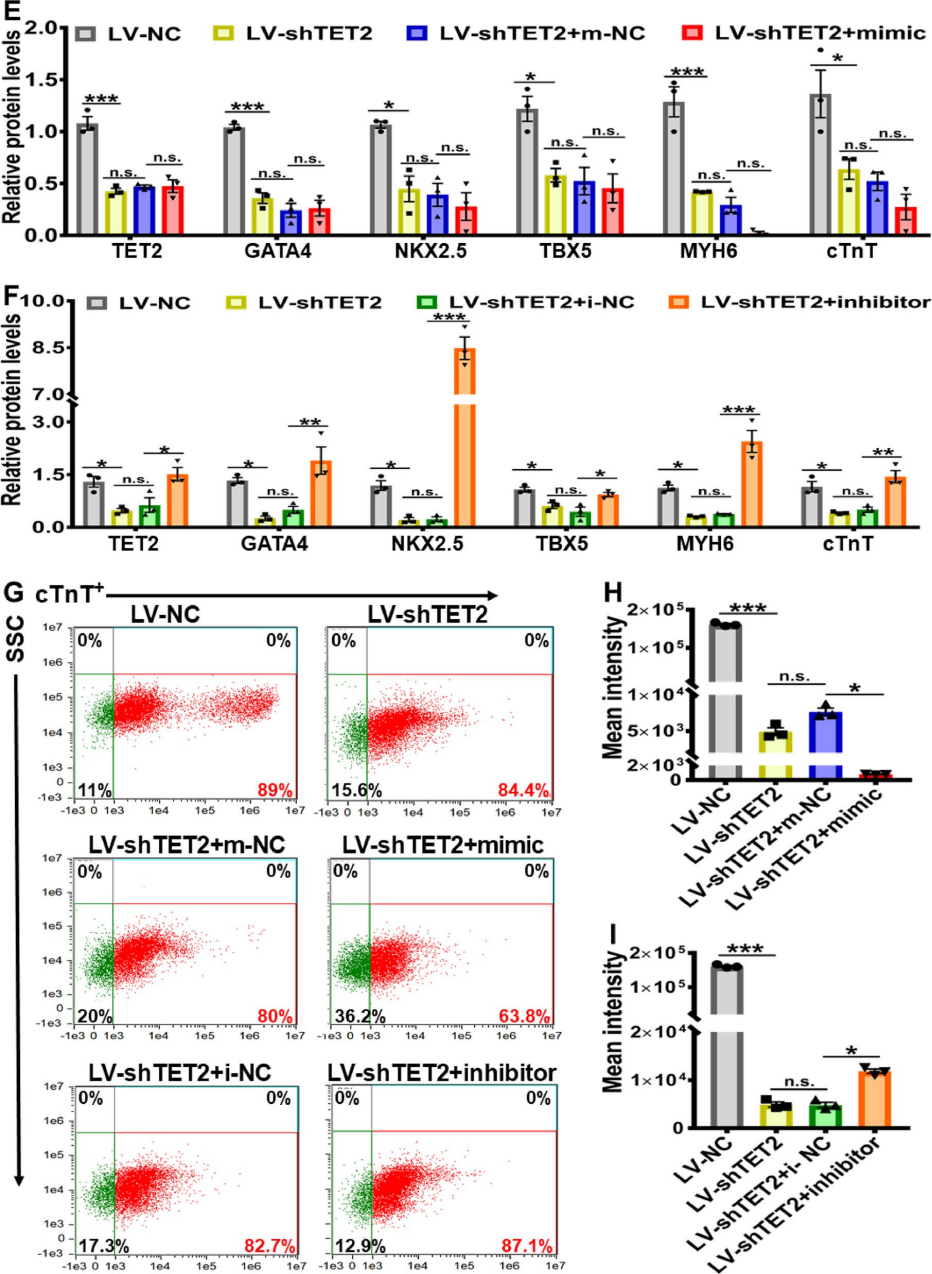

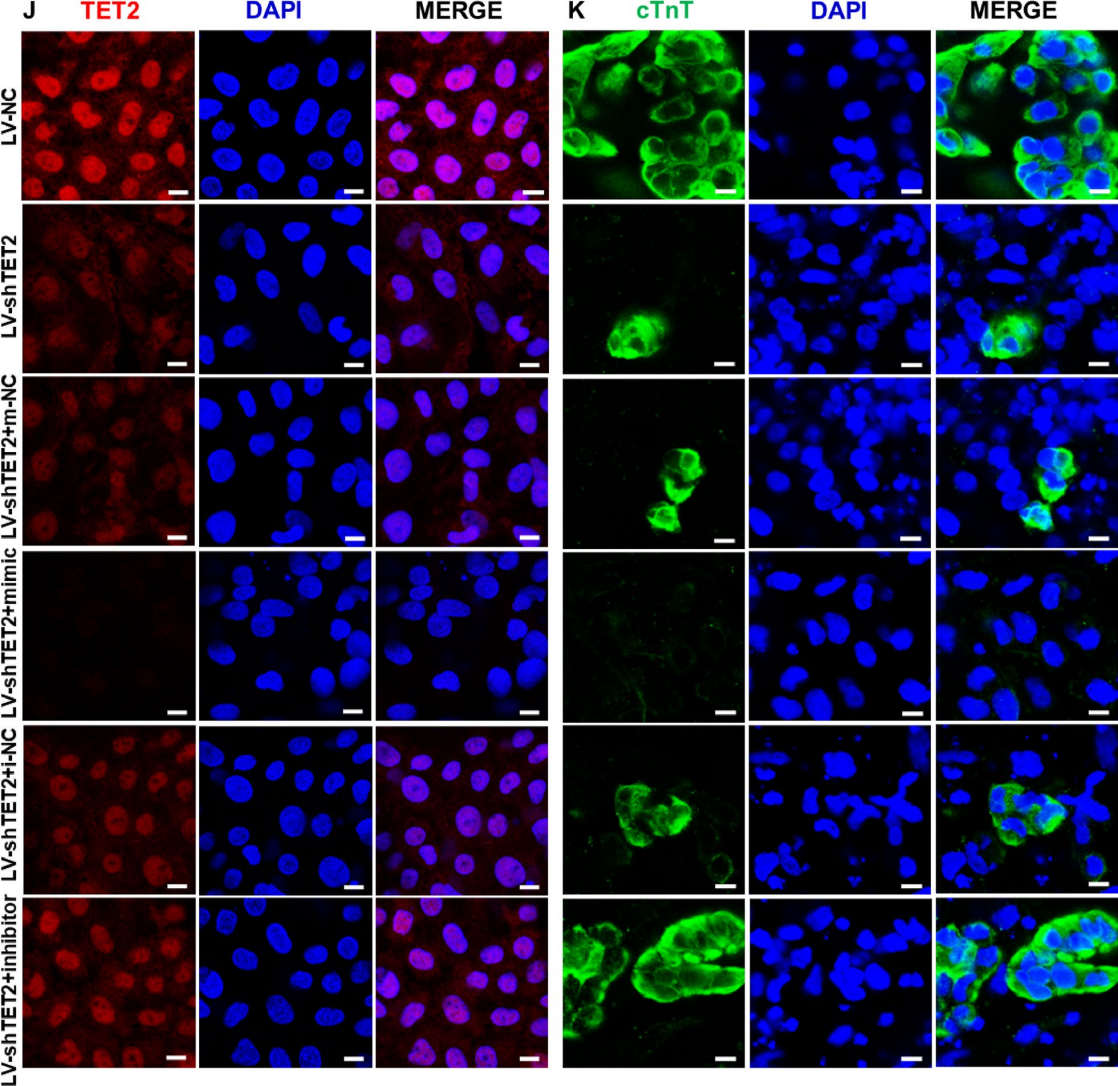
Reversal of TET2 knockdown-mediated suppression in hESCs-derived cardiac differentiation by inhibiting miR-20b-5p. **A** qRT-PCR analysis of the relative mRNA expressions levels of *TET2* and cardiac transcriptional factors (*GATA4*, *NKX2.5*, *TBX5*, *MYH6* and *cTnT*) in 12-day cardiac-differentiated hESCs with TET2 knockdown and transfection of m-NC (mimic-negative control) or miR-20b-5p-mimic (*n* = 3). **B** qRT-PCR analysis of the relative mRNA expressions levels of *TET2* and cardiac transcriptional factors (*GATA4*, *NKX2.5*, *TBX5*, *MYH6* and *cTnT*) in 12-day cardiac-differentiated hESCs with TET2 knockdown and transfection of i-NC (inhibitor-negative control) or miR-20b-5p- inhibitor (*n* = 3). **C **and** E** Western blot analysis **C** and quantification **E** of the relative protein levels of TET2 and cardiac transcriptional factors (GATA4, NKX2.5, TBX5, MYH6 and cTnT) in 12-day cardiac-differentiated hESCs with TET2 knockdown and transfection of m-NC or miR-20b-5p-mimic (*n* = 3). **D** and** F** Western blot analysis **D** and quantification **F** of the relative protein levels of TET2 and cardiac transcriptional factors (GATA4, NKX2.5, TBX5, MYH6 and cTnT) in 12-day cardiac-differentiated hESCs with TET2 knockdown and transfection of i-NC or miR-20b-5p-inhibitor (*n* = 3). **G-I** Flow cytometry analysis **G** and quantification **H** and** I** of cTnT-positive cell in 12-day cardiac-differentiated hESCs with TET2 knockdown and transfection of m-NC, miR-20b-5p-mimic, i-NC or miR-20b-5p-inhibitor (*n* = 3). **J** and** K** Immunofluorescence staining of TET2 **J** and cTnT **K** in 12-day cardiac-differentiated hESCs with TET2 knockdown and transfection of m-NC, miR-20b-5p-mimic, i-NC or miR-20b-5p-inhibitor (Scale bar = 10 μm). Quantitative data were presented as mean ± SEM. Statistical significance was analyzed via a one-way ANOVA followed by Bonferroni multiple comparisons test and represented as *P < 0.05, **P < 0.01 and ***P < 0.001, while n.s. indicated non-significance

**Fig. 8:**
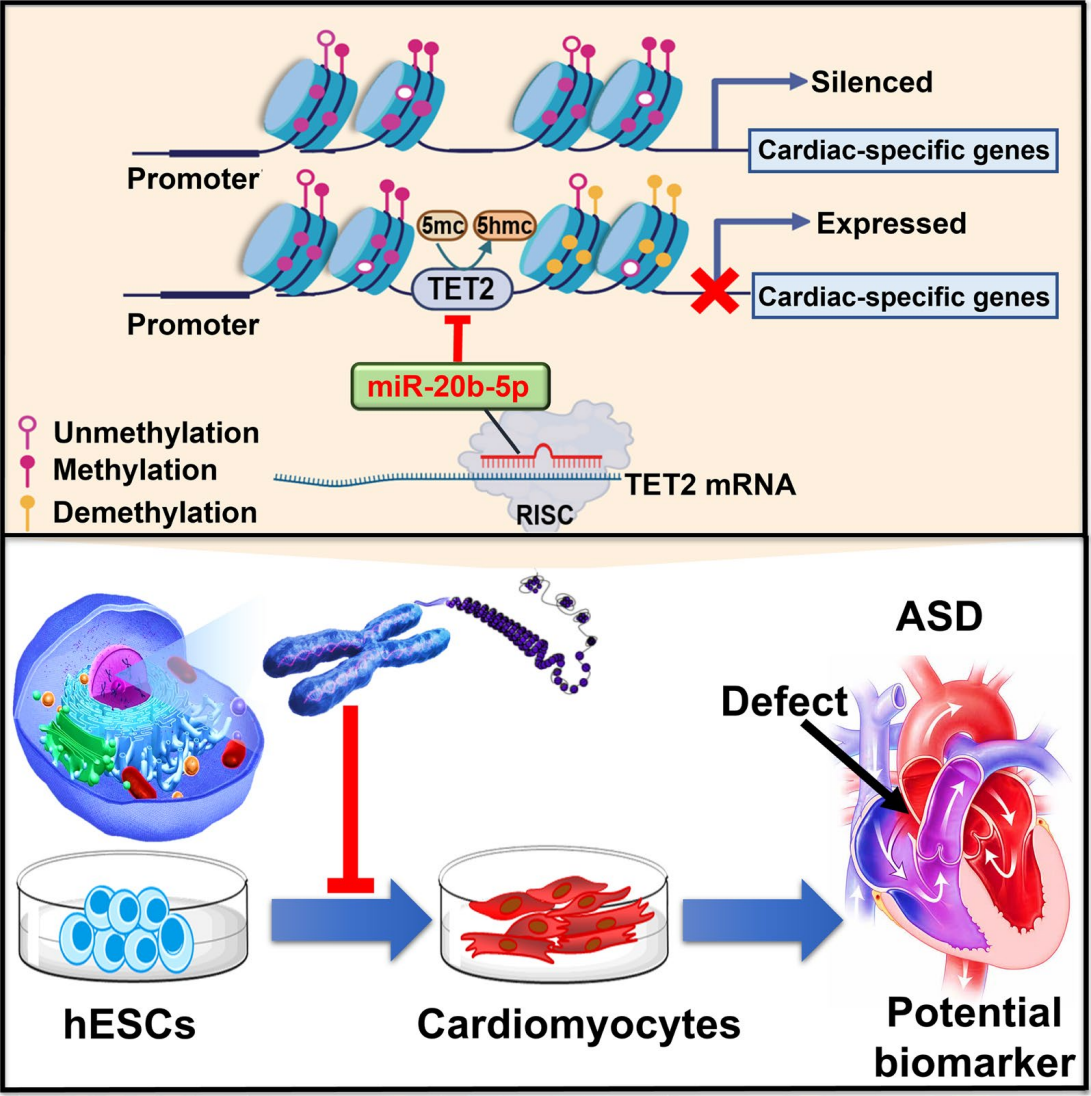
Graphic illustration of proposed mechanism of miR-20b-5p/TET2 axis in cardiac differentiation. The epigenetic mechanism of miR-20b-5p/TET2 axis in cardiac differentiation is illustrated whereby miR-20b-5p acts as an inhibitory regulator in hESCs-derived cardiac differentiation by targeting TET2, leading to suppression of 5-hydroxymethylcytosine and cardiac-specific genes expression. These findings indicate that miR-20b-5p could be a potential biomarker for ASD

As demonstrated in Fig. 7A and B, knockdown of TET2 (LV-shTET2) led to a significant decrease in the expression of key cardiac transcription factors in 12-day cardiac-differentiated hESCs, including *GATA4*, *NKX2.5*, *TBX5*, *MYH6* and *cTnT*, when compared to the LV-NC group. Remarkably, treatment with the miR-20b-5p mimic in LV-shTET2 group led to a further reduction in the mRNA expression of these cardiac transcriptional factors in 12-day cardiac-differentiated hESCs, comparing to the LV-NC group (Fig. 7A). On the contrary, exposure to the miR-20b-5p inhibitor resulted in a significant increase in the mRNA expression level of these cardiac genes in the LV-shTET2 group (Fig. 7B). Due to the effective suppression of cardiac differentiation by TET2 knockdown in the LV-shTET2 group, additional treatment with the miR-20b-5p mimic did not induce a significant change in the protein level of these cardiac genes in the LV-shTET2 group (Fig. 7C and E). However, a significantly upregulated protein level of these cardiac genes was consistently detected in the LV-shTET2 group after exposure to the miR-20b-5p inhibitor (Fig. 7D and F). Interestingly, flow cytometry analysis revealed that TET2 knockdown by LV-shTET2 caused an insignificant effect on the percentage of cTnT-positive cells after 12-day cardiac differentiation of hESCs in the LV-shTET2 group, when compared to the LV-NC group (Fig. 7G). Nevertheless, the markedly decreased mean fluorescence intensity in 12-day differentiated hESCs-CM was detected in LV-shTET2 group compared to LV-NC group (Fig. 7H). Furthermore, in the cardiac differentiation of TET2 knockdown hESCs, treatment with the miR-20b-5p mimic resulted in a decrease in both the percentage of cTnT-positive cells and the mean fluorescence intensity, in comparison with the mimic-NC group (Fig. 7G and H). On the other hand, exposure to the miR-20b-5p inhibitor caused a notable increase in mean fluorescence intensity compared with the inhibitor-NC group (Fig. 7I). Consistently, immunofluorescence analysis confirmed the downregulation of both TET2 and cTnT in 12-day cardiac-differentiated hESCs with transfection of LV-shTET2, and additional treatment with the miR-20b-5p mimic caused a further decrease in the expression of TET2 and cTnT in the LV-shTET2 group (Fig. 7J and K). Conversely, additional administration of the miR-20b-5p inhibitor significantly reversed the inhibitory expression of TET2 and cTnT in the LV-shTET2 group (Fig. 7J and K). These findings suggest that inhibition of miR-20b-5p can partially reverse the suppressive effect of TET2 knockdown on the cardiac differentiation of hESCs, supporting the proposed mechanism whereby miR-20b-5p acts as a crucial regulator in inhibition of cardiac differentiation via targeting TET2 (Fig. 8).

## Discussion

Congenital heart disease is prevalent congenital heart malformations with severe cardiac dysfunction, which are related to the complex regulation of cardiac transcription factors involving multiple genetic and epigenetic regulators during heart development [33]. Epigenetics, such as DNA methylation and hydroxymethylation, are crucial regulators of CHD development. In Particular, TET2, the primary DNA-modifying enzyme for DNA hydroxymethylation, has been shown to play a significant role in modulating cardiogenesis by activating specific cardiac transcription factors [34]. In addition, recent evidence has demonstrated that miRNAs are vital regulators in of CHD development [35–37]. However, the specific role of miRNAs in regulating TET2 during cardiac development and CHD pathogenesis remains unknown, and the underlying epigenetic mechanism is unclear.

In this study, miRNA profiling revealed that miR-20b-5p was remarkably decreased during 12-day cardiac differentiation of hESCs but increased in plasma samples from ASD patients. It was further demonstrated that miR-20b-5p repressed hESCs-derived cardiac differentiation by downregulating key cardiac transcription factors (*GATA4*, *NKX2.5*, *TBX5*, *MYH6* and *cTnT*), which was directly mediated by miR-20b-5p/TET2 axis as a negative regulator in cardiac differentiation. Reversely, the treatment of miR-20b-5p inhibitor restored the inhibition of hESCs-derived cardiac differentiation caused by TET2 knockdown. Importantly, this study identifies the inhibitory role of miR-20b-5p/TET2 axis in cardiac differentiation for the first time, providing experimental evidence that miR-20b-5p could serve as a promising regulator and biomarker for ASD.

MiRNAs have emerged as potential biomarkers and therapeutic targets for various cardiovascular diseases, including several subtypes of CHD. For example, a total of four miRNAs including miR-19b, miR-29c, miR-22 and miR-375 were found to increase in TOF, while miR-19b and miR-29c were also upregulated in VSD [38, 39]. Therefore, we conducted RNA-sequencing analysis to identify miRNA expression profiles in both in vitro cardiac differentiation of hESCs and plasma samples from ASD patients. In the cardiac differentiation model of hESCs, 46 miRNAs were found to be downregulated, while 48 miRNAs were upregulated at each time point (D0, D3, D6, D9 and D12) during the 12-day differentiation process. KEGG pathway analysis revealed that these miRNAs were enriched in pathways related to cardiac development, including pluripotency signaling, Hippo signaling and Wnt signaling, which were consistent with previous findings indicating the critical roles of Hippo and Wnt signaling pathways in heart growth, cardiomyocyte proliferation, survival and commitment [40–42]. Integration of RNA-sequencing analysis from 12-day cardiac-differentiated hESCs and plasma samples from ASD patients led to the identification of eight candidate miRNAs exhibiting reduced expression levels in cardiac-differentiated hESCs but elevated expression profiles in plasma samples from ASD patients. These candidate miRNAs include hsa-miR-20b-5p, hsa-miR-3173-5p, hsa-miR-222-5p, hsa-miR-335-3p, hsa-miR-378a-5p, hsa-miR-4746-5p, hsa-miR-486-5p and hsa-miR-92b-3p. Subsequently, our study evaluated the potential role of these eight candidate miRNAs in cardiac differentiation, as well as their potential epigenetic mechanisms.

Several studies have emphasized the significance of TET2 and DNA hydroxymethylation in cardiogenesis [43]. TET2 has been shown to trigger the activation of specific transcription factors (TMEM88 and NKX2.5) in cardiac differentiation [44]. Conversely, loss of TET2 was found to disrupt the epigenetic landscape and alter chromatin accessibility, thereby preventing cardiac gene expression during embryonic heart development [27]. Consistent with these reports, our findings also illustrated a time-dependent upregulation of TET2 and 5hmC during in vitro cardiac differentiation models such as hESCs-CMs, hiPSCs-CMs and mESCs-CMs, as well as in vivo embryonic and neonatal mouse hearts. Furthermore, our study confirmed that knockdown of TET2 significantly suppressed hESCs-derived cardiac differentiation by inhibiting the expression of key cardiac transcription factors such as GATA4, NKX2.5, TBX5, MYH6 and cTnT. Our results are in line with previous studies showing the role of TET2 in promoting cardiac differentiation. Consequently, we further determined whether the eight candidate miRNAs identified from the cardiac-differentiated hESCs and the plasma samples of ASD patients have the potential to target TET2 using the TargetScan database. Intriguingly, among the eight candidate miRNAs, only miR-20b-5p was predicted to target TET2 with a high probability, indicating the potential involvement of miR-20b-5p/TET2 axis in cardiac differentiation and ASD pathogenesis. MiR-20b-5p has been reported to involve in various diseases, including hypoxia/reoxygenation-stimulated cardiac autophagy and apoptosis, diabetic impaired wound healing, diabetic retinal vascular dysfunction and Alzheimer's [45–48]. However, the role of miR-20b-5p in cardiac differentiation and ASD pathogenesis has not been previously reported, suggesting the novel founding of miR-20b-5p as a potential regulator for cardiogenesis.

It has demonstrated the significance of miRNAs in modulating cardiogenesis by interacting with multiple cardiac transcription factors. For instance, deletion of the MIR148A family resulted in a reduced proportion of cardiomyocytes after cardiac induction [49]. Moreover, miR-335-3p/5p has been found to potentially upregulate cardiac mesoderm marker (BRACHYURY) and cardiac progenitor cells markers (GATA4 and NKX2.5) by activating WNT and TGFβ signaling pathways [50]. Despite extensive studies of miRNAs involving cardiac differentiation, the role of miR-20b-5p in this process remains largely obscure. While the essential role of TET2 in cardiac differentiation has been established in both previous reports and our in vitro results, the impact of miR-20b-5p on TET2 during cardiac differentiation remains unknown. Thus, this study aimed to investigate the role of miR-20b-5p during 12-day cardiac differentiation of hESCs, with a specific focus on its association with TET2 and DNA hydroxymethylation. Our results demonstrated that a time-dependent downregulation of miR-20b-5p in several in vitro cardiac differentiation models, including 12-day differentiation of hESCs-CMs, hiPSCs-CMs and mESCs-CMs, as well as in vivo embryonic and neonatal mouse hearts. Using gain or loss-of-function models with miR-20b-5p mimic or inhibitor, we further revealed that the treatment with miR-20b-5p mimic significantly decreased the proportion of cTnT-positive cells and the expression of key cardiac transcriptional factors, including GATA4, NKX2.5, TBX5, MYH6 and cTnT. Conversely, exposure to miR-20b-5p inhibitor led to positive regulation on hESC-derived cardiac differentiation. More importantly, administration of miR-20b-5p mimic reduced TET2 and 5hmC levels as well as the proportion of TET2-positive cells in 12-day cardiac-differentiated hESCs, while treatment with miR-20b-5p inhibitor reversed the inhibitory effect of TET2 in hESCs-derived cardiac differentiation. Additionally, the direct targeting of miR-20b-5p to the 3'UTR of TET2 mRNA was confirmed by the dual luciferase reporter assay, which is consistent with our results previously predicted by TargetScan**.** These findings preliminarily verify that miR-20b-5p negatively regulates TET2 in hESCs-derived cardiac differentiation.

To further elucidate the inhibitory effect of miR-20b-5p on TET2 during cardiac differentiation, miR-20b-5p mimic and inhibitor were administrated to TET2-knowndown hESCs during 12-day cardiac differentiation. Inspiringly, our results also confirmed the suppressive effect of miR-20b-5p mimic and the enhancing effect of miR-20b-5p inhibitor on the mRNA expression of key cardiac genes and the percentage of cTnT-positive cells in TET2-knowndown hESCs after 12-day cardiac differentiation. Besides, our findings demonstrated that inhibition of miR-20b-5p significantly reversed the suppressive effect of TET2 knockdown on hESCs-derived cardiac differentiation, thereby supporting the proposed mechanism that miR-20b-5p acts as a negative regulator of hESCs-derived cardiac differentiation by targeting TET2. Overall, the current study offers insight into the direct repressive effect of miR-20b-5p on in vitro cardiac differentiation of hESCs through targeting TET2. Finally, the epigenetic mechanism of miR-20b-5p/TET2 axis in modulating in vivo cardiogenesis and ASD pathogenesis is far from elucidated, and further investigation is required to clarify the influence of miR-20b-5p on TET2 and in vivo cardiac development.

## Conclusions

In conclusion, this study demonstrates that miR-20b-5p functions as an inhibitory regulator in hESCs-derived cardiac differentiation by targeting TET2. Our results illustrate a novel epigenetic mechanism of miR-20b-5p/TET2 axis in cardiac differentiation, which sheds light on the intricate epigenetic regulation in cardiac differentiation. Although the effects of miR-20b-5p on ASD needs to be further elucidated, this study extends our insight into the role of miR-20b-5p/TET2 axis in suppression of cardiac development, which provides a potential biomarker for ASD.

## Methods

### Stem cells maintenance and cardiac differentiation

The H1 cell lines of hESCs and hiPSCs were maintained on Matrigel-coated plates with mTeSR1 medium (Stem Cell Technologies, Canada) for 3 days till 80% confluence. To initiate cardiac differentiation, the cells were cultured with a series of cardiac differentiation medium (I, II and III) for 12 days according to the manufacturer’s protocols (CELLAPY, China). Meanwhile, the maintenance and cardiac differentiation of mESCs were performed as previously described [22]. Briefly, the mESCs were seeded onto gelatin-coated plates and cultured in high glucose DMEM medium (GIBCO, USA) supplemented with 10% FBS (ExCell Bio, China) and 1,000 U /ml of leukemia inhibitory factor (LIF) (Millipore, USA). To generate embryoid bodies (EBs), mESCs were developed into EBs (1,250 cells/drop) in a 3D hanging-drop culture for 4 days with LIF-free medium. The early EBs were later transferred to gelatin-coated 12-well culture plates for 8-day adherent differentiation with LIF-free medium.

### Specimen collection and ethical statement

This study was approved by the Ethics Committee of Guangdong Provincial People's Hospital (KY-Q-2021-277-01) and was performed in accordance with Helsinki declaration. In brief, peripheral blood samples were collected from 10 ASD patients and 9 healthy controls aged from 2 to 8 years, with written informed consent obtained from the parents of each participant. Whole transcriptome sequencing was later performed on plasma extracted from these peripheral blood samples. The comparison of the age and gender between the two groups did not reveal significant difference (Additional file 1: Table S7). The ASD patients were recruited from Guangdong Provincial People's Hospital and confirmed by echocardiography, while healthy controls were from the community. None of the participants had any other underlying health conditions.

### MicroRNA expression profiling

The total RNA was extracted from cardiac-differentiated hESCs using TRIzol (Invitrogen, USA) in accordance with the manufacturer's protocol. The quantity and quality of small RNA were evaluated using RiboBio Co., Ltd. The yield and integrity of the RNA were then determined by Qubit®2.0 (Life Technologies, USA) and Agilent 2200 TapeStation (Agilent Technologies, USA), respectively. Subsequently, 1 µg of RNA from each sample was employed for the preparation of small RNA libraries using NEBNext® Multiplex Small RNA Library Prep Set for Illumina (NEB, USA) according to the manufacturer's instructions. The libraries were then sequenced using a HiSeq 2500 (Illumina, USA) platform with a single end 50 bp configuration by RiboBio Co., Ltd. (RiboBio, China). The differential expression of miRNAs between groups was calculated using the edgeR algorithm, with the criteria of **|** log2(Fold Change) **|**≥ 1 and *P*-value < 0.05. Gene ontology (GO) and Kyoto Encyclopedia of Genes and Genomes (KEGG) pathway analysis were further carried out using the KOBAS tool.

Total RNA in plasma samples were extracted from peripheral blood samples collected from ASD patients or healthy individuals using TRIzol® Reagent (Plant RNA Purification Reagent for plant tissue) according to the manufacturer's guidelines (Invitrogen, USA). The quality of extracted RNA was assessed using 2100 Bioanalyzer (Agilent Technologies, USA) and quantified with ND-2000 (NanoDrop Technologies, USA). The RNA purification, reverse transcription, library construction and sequencing procedures were carried out at Shanghai Majorbio Bio-pharm Biotechnology Co., Ltd. (Shanghai, China), in accordance with the manufacturer's instructions (Illumina, San Diego, CA). A total of 3 µg of total RNA per sample was utilized as the input material for the small RNA library. The sequencing libraries were generated using the Truseq TM Small RNA sample prep Kit from Illumina (San Diego, CA) in compliance with the manufacturer's recommendations. The differential expression of miRNAs between the two sets of samples was calculated using the NOIseq algorithm, with the criteria of Prob value > 0.8.

### Animal study and ethical statement

The animal experiments were approved by the Animal Research Committee of Guangzhou Medical University (GY2021-054) and the procedures conformed to the National Institutes of Health (NIH) Guide for the Care and Use of Laboratory Animals. C57BL/6 J mice (8 weeks old) were purchased from the Medical Experimental Animal Center of Guangdong Province. All the mice were raised with food and water ad libitum and housed in humidity and temperature-controlled environment with a 12/12-h light/dark cycle. Female and male mice were mated in cages, and vaginal plug was monitored every morning. Once the vaginal plug was observed, it was designated as embryonic day 0.5 (E0.5). Besides, the first postnatal day was designated as postnatal day 0 (P0). Eventually, the mice were euthanized by intraperitoneal injection of 200 mg/kg pentobarbital. The heart samples from either the embryos or neonates were collected on certain embryonic or postnatal day according to experimental requirements.

### Knockdown of TET2 in hESCs

A lentivirus vector (LV)-shTET2 with enhanced green fluorescent protein (eGFP) was obtained from Vector Builder (Guangzhou, China), while an empty vector with eGFP was utilized as a negative control (LV-NC). hESCs were cultured in 12-well plates till 50% confluence and then infected with lentivirus particles in the presence of 5 μg/ml polybrene at a multiplicity of infection of 50. The cells were maintained for a minimum of 4 days and then screened for puromycin resistance. The knockdown efficiency of TET2 was evaluated through qRT-PCR and western blotting. The primers used for the shRNA target sequences are listed in Additional file 1: Table S8.

### MicroRNA transfection

The agents including negative control of mimic (m-NC), miR-20b-5p mimic, negative control of inhibitor (i-NC) and miR-20b-5p inhibitor were obtained from RiboBio (Guangzhou, China). To investigate the role of miR-20b-5p in hESCs-derived cardiac differentiation, the hESCs were subjected to 12-day cardiac differentiation with transfection of either 100 nM of m-NC or miR-20b-5p mimic, 300 nM of i-NC or miR-20b-5p inhibitor using the miRNA transfection reagent (RiboBio, China) in accordance with the manufacturer's instructions. The transfection was carried out bi-daily throughout the 12-day process of cardiac differentiation.

### Western blotting

Total protein was extracted from cell samples or heart tissues using a protein lysis buffer containing protease and phosphatase inhibitors (Beyotime, China). The protein concentration was then determined using a Bicinchoninic Acid protein assay kit (Thermo Fisher Scientific, USA). The proteins were separated using either a 6% or 12% Sodium dodecyl sulfate–polyacrylamide gel electrophoresis (Beyotime, China) and transferred onto polyvinylidene difluoride membranes (Millipore, USA). To block non-specific binding, the membranes were incubated in 5% non-fat milk for 1 h. Subsequently, the membranes were incubated overnight at 4 °C with primary antibodies including rabbit anti-α-tubulin (diluted 1:1000, Abcam, ab52866), rabbit anti-GAPDH (diluted 1:1000, Cell Signaling Technology, #5174), rabbit anti-TET2 (diluted 1:1000, Cell Signaling Technology, #45010), rabbit anti-TBX5 (diluted 1:1000, Abcam, ab137833), mouse anti-NKX2-5 (diluted 1:1000, Abcam, ab91196), rabbit anti-GATA4 (diluted 1:1000, Abcam, ab134057), rabbit anti-cTnT (diluted 1:1000, Abcam, ab209813) and mouse anti-MYH6 (diluted 1:1000, Abcam, ab50967). The membranes were then incubated with horseradish peroxidase-conjugated secondary antibodies (diluted 1:5000, ZSGB-Bio, ZB-2301, ZB-2305) for 1 h at room temperature. Antibody binding was visualized using an ECL detection reagent (Beyotime, China) and captured with Amersham Imager 600 (GE Healthcare, USA). Density analysis of the bands was performed using Image J, and the relative protein levels were normalized to the expression of either GAPDH or α-tubulin.

### Quantitative real time-PCR (qRT-PCR)

Total RNA was extracted from cell samples or heart tissues using TRIzol reagent (Accurate Biology, China). Complementary DNA (cDNA) was then synthesized by a reverse transcription kit (Accurate Biology, China) according to the manufacturer's protocol. qRT-PCR was carried out on the Lightcycler480 system (Roche, USA) using SYBR Green PCR Kit (Accurate Biology, China). The level of miR-20b-5p was detected using stem-loop primers obtained from RiboBio (Guangzhou, China), while other gene primers used for qRT-PCR were obtained from Tsingke Biotechnology (Beijing, China). The results were normalized to the expression of either U6 or GAPDH, and relative fold changes were calculated using the 2^−△△Ct^ method. The primers used for qRT-PCR are listed in Additional file 1: Table S9.

### Immunofluorescence staining

The cardiac-differentiated hESCs were fixed with 4% paraformaldehyde (Biosharp, China) for 20 min, followed by washing with PBS. The cells were then permeabilized with 0.25% Triton X-100 for 20 min, followed by washing with PBS. The cells were blocked with 10% goat serum for 1 h at room temperature and then incubated with primary antibodies overnight at 4 °C. The primary antibodies included rabbit anti-TET2 (diluted 1:200, Invitrogen, PA5-85488) and rabbit anti-cTnT (diluted 1:200, Abcam, ab209813). After washing with PBS, the cells were incubated with secondary antibodies at room temperature for 1 h. The secondary antibodies included Anti-rabbit IgG (H + L), F(ab’)2 Fragment (Alexa Fluor® 488 Conjugate) (diluted 1:800, Cell Signaling Technology, #4412) and Anti-Rabbit IgG (H + L) Cross-Adsorbed Secondary Antibody (Alexa Fluor™ 647) (diluted 1:800 Invitrogen, A-21244). The nuclei were then stained with DAPI (Beyotime, C1002), and fluorescence images were obtained by a laser scanning confocal microscope (LSM 880, Carl Zeiss).

### Flow cytometry analysis

The cardiac-differentiated hESCs were isolated and analyzed by flow cytometry. Approximately 1 × 10^5^ cells per sample were fixed with 4% paraformaldehyde (Biosharp, China) and washed with staining buffer (1% fetal bovine serum in PBS) (Beyotime, China). The cells were then permeabilized with 100% cold methanol and incubated with primary antibodies, followed by the corresponding secondary antibody. The primary antibodies contained rabbit anti-TET2 (diluted 1:500, Abcam, ab94580) and rabbit anti-cTnT (diluted 1:500, Abcam, ab209813). The secondary antibodies used were Anti-rabbit IgG (H + L), F(ab’)2 Fragment (Alexa Fluor® 488 Conjugate) (diluted 1:1000, Cell Signaling Technology, #4412) and Anti-Rabbit IgG (H + L) Cross-Adsorbed Secondary Antibody (Alexa Fluor™ 647) (diluted 1:1000, Invitrogen, A-21244). As shown in Additional file 1: Fig. S6, cell gating was performed by using isotype controls, such as Rabbit (DA1E) mAb IgG XP® Isotype Control (Alexa Fluor® 647 Conjugate) (diluted 1:1000, Cell Signaling Technology, #2985) and Rabbit (DA1E) mAb IgG XP® Isotype Control (Alexa Fluor® 488 Conjugate) (diluted 1:1000, Cell Signaling Technology, #2975). The samples were acquired using Amnis Image StreamX MarkII flow cytometer (Merck & Millipore, Germany) and analyzed with IDEAs.6.2 software.

### Dot blot analysis

The detection of 5-hydroxymethylcytosine (5hmC) was performed by dot blot analysis. The genomic DNA was isolated by the DNA Purification Kit (TIANGEN, China) and was then diluted with TE buffer. The DNA samples were subjected to denaturation at 95 °C for 10 min, followed by rapid cooling on ice. The denatured samples were applied onto a nitrocellulose membrane (Biosharp, China) which had been pre-wetted. The membrane was washed for 10 min in 2 × SSC solution, dried for 10 min at room temperature and exposed to ultraviolet (UV) radiation for 15 min. The membrane was then blocked with 3% bovine serum albumin-TBST (BSA-TBST) for 1 h at room temperature (Beyotime, China) and incubated with mouse anti-5hmC antibody (diluted 1:1000, Cell Signaling Technology, #51660) at 4 °C overnight. Subsequently, the membrane was incubated with HRP-conjugated anti-mouse IgG secondary antibody (diluted 1:2000, ZSGB-Bio, ZB-2305) for 1 h at room temperature. The antibody binding was visualized using ECL detection reagent (Beyotime, China), and digital images were captured using Amersham Imager 600 (GE Healthcare, USA). Methylene blue staining of the membranes was used to confirm equal DNA loading. Finally, the densitometry of the bands was quantified by Image J.

### Dual luciferase reporter assay

HEK-293 T cells were seeded into a 96-well plate and allowed to reach 50–70% confluence prior to transfection. The pmiR-RB-Report™ luciferase vector (RiboBio, China) was modified by the introduction of either wild-type or mutant TET2 fragments (524 bp) into its restriction sites and designated as LUC-WT-TET2 or LUC-MUT-TET2, respectively. The transfection was performed using lipofectamine 3000 (Invitrogen, USA) with 150 ng of plasmids of LUC-WT-TET2 and LUC-MUT-TET2, and then exposed to 100 nM of m-NC and miR-20b-5p mimic, 300 nM of i-NC and miR-20b-5p inhibitor. The culture medium was replaced 6 h post-transfection. After 48-h incubation, the Dual-Luciferase system (Promega, USA) was utilized to determine the firefly and Renilla luciferase activities. The medium was aspirated, and the cells were treated with 50 μL of PBS and luciferase reagent. The 96-well plate was then incubated on a shaker at room temperature for 10 min before the determination of firefly luciferase activity. The Renilla luciferase activity was determined by adding 30 μL of stop reagent per well and shaking for 10 min. The luciferase activities were then detected using Agilent BioTek Synergy LX. Finally, the differences between firefly and Renilla luciferase activities were calculated to determine the relative luciferase activity.

### Statistical analysis

Quantitative data were demonstrated as means ± SEM. A two-tailed unpaired Student’s t test was used for analysis of two-group difference, while a one-way ANOVA followed by Bonferroni multiple comparisons test was introduced for assessment of multiple group variations. The differences were considered to be statistically significant, when *P < 0.05, **P < 0.01 and ***P < 0.001.

### Supplementary Information

**Additional file1**. Supplementary materials include supplemental figures 1-6 and supplemental tables 1-9.

**Additional file2**. Video of spontaneously beating hESCs after 9-day cardiac differentiation.

## Data Availability

The datasets used and/or analyzed during the current study are available from the corresponding author on reasonable request.
