# Supplementary material for: Identification of miR-20b-5p as an inhibitory regulator in cardiac differentiation via TET2 and DNA hydroxymethylation
Source: Clin Epigenetics. 2024 Mar 15;16:42. doi: 10.1186/s13148-024-01653-7 (PMC10943922; doi:10.1186/s13148-024-01653-7)
Supplement: Supplementary file 1 — Additional file1. Supplementary materials include supplemental figures 1-6 and supplemental tables 1-9. [file 13148_2024_1653_MOESM1_ESM.docx]

**Supplementary Information**

***Of***

**Identification of MiR-20b-5p as an Inhibitory Regulator in Cardiac Differentiation via TET2 and DNA Hydroxymethylation**

**Supplemental figures and figure legends**

**
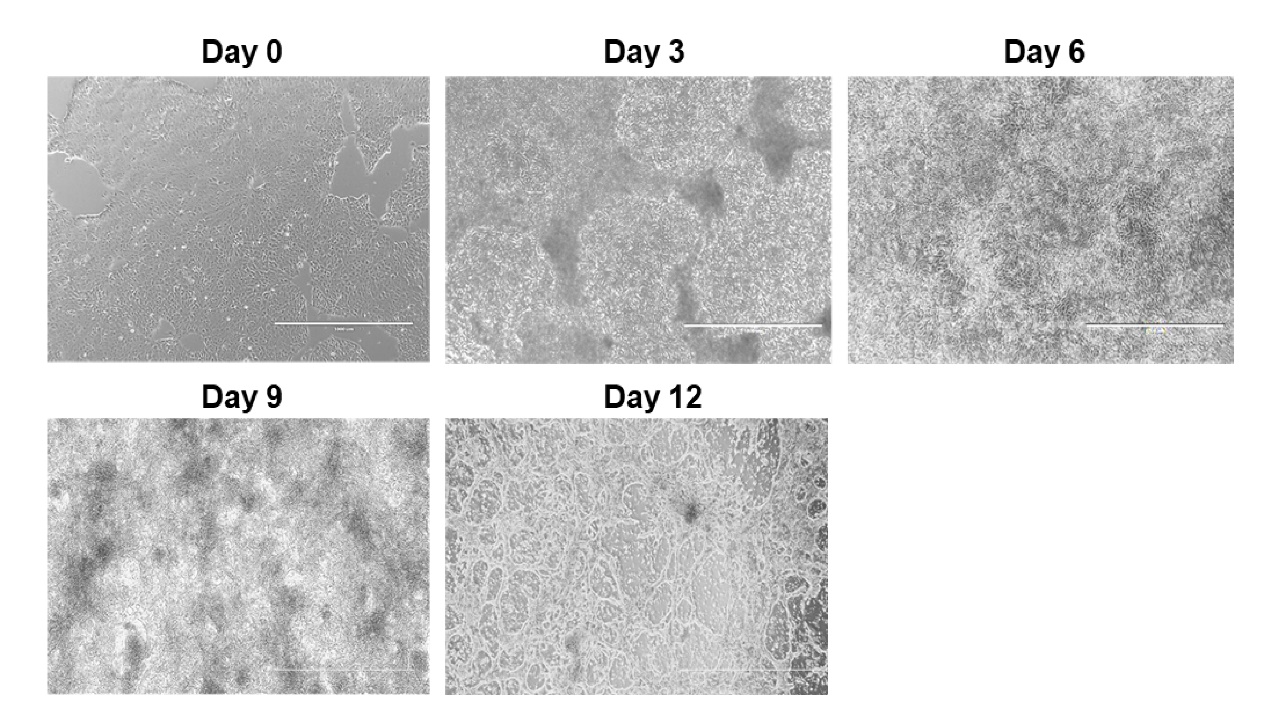
**

**Figure S1 Morphological changes of hESCs during 12-day cardiac differentiation (Scale bar = 1000 μm).**

**Video 1 Spontaneously beating hESCs after 9-day cardiac differentiation.**


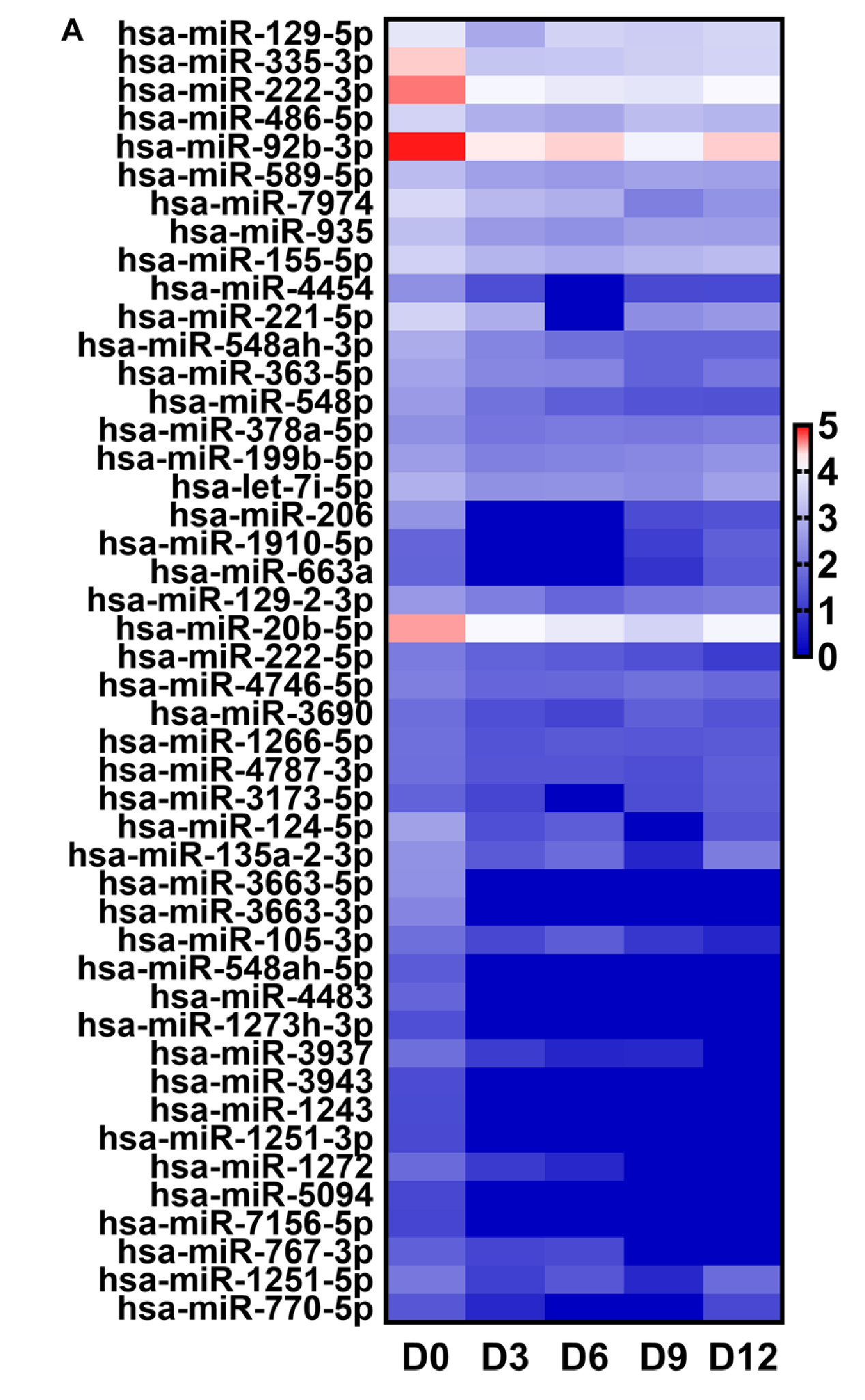


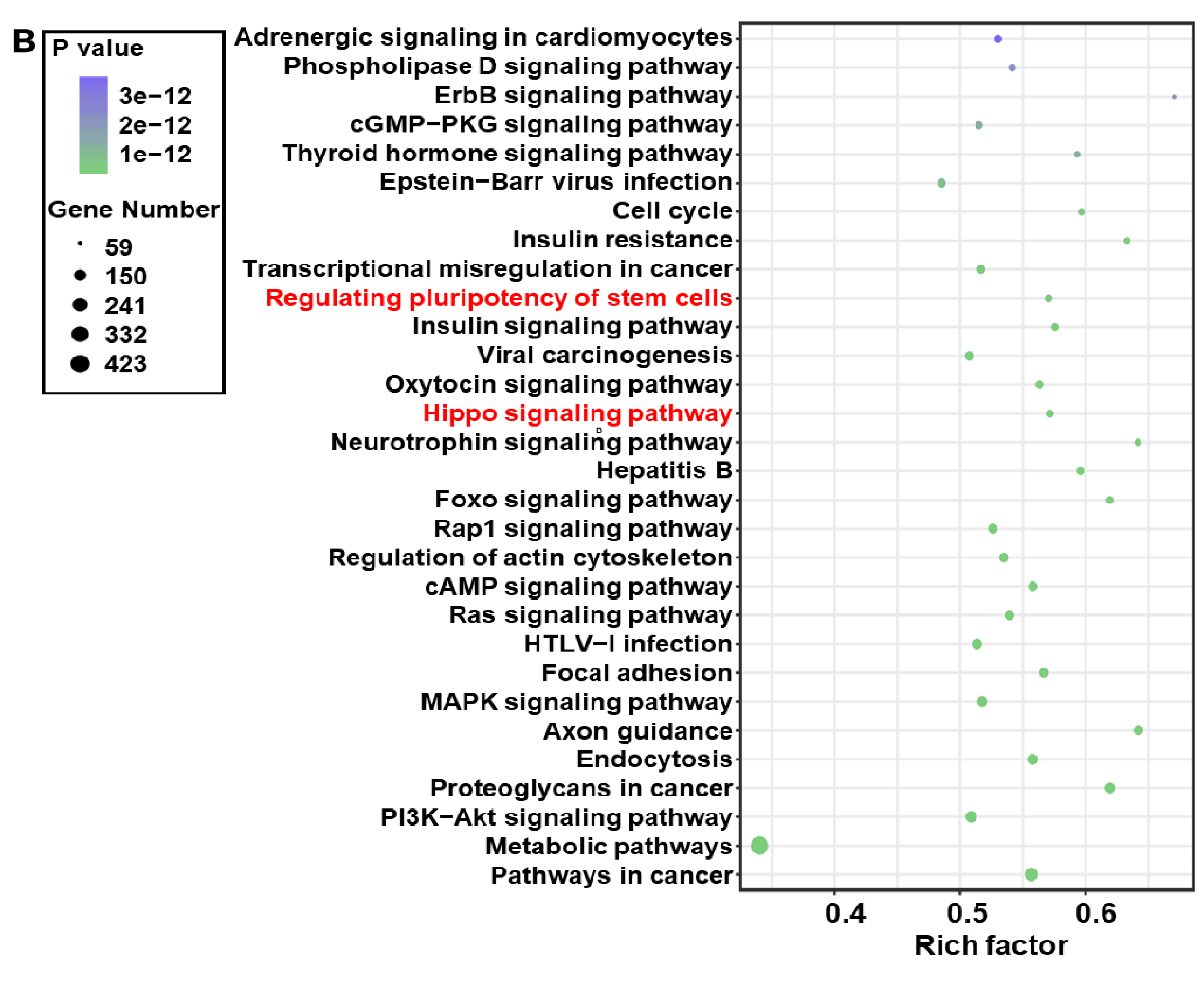


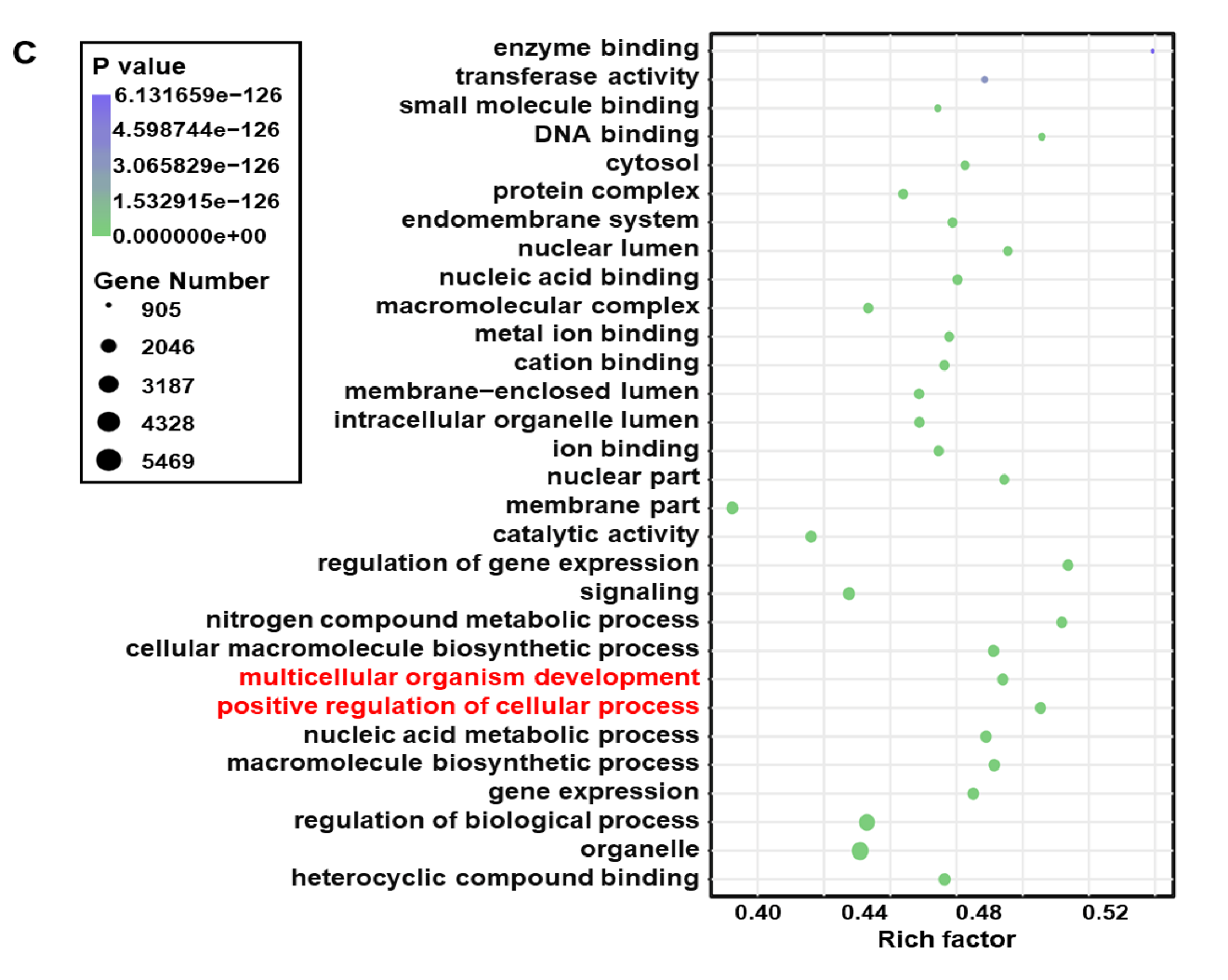


**Figure S2** **RNA sequencing with KEGG and GO analysis of down-regulated miRNAs during 12-day hESCs-derived cardiac differentiation.**

**(A)** Heatmap displaying the expression levels of 46 miRNAs downregulated in hESCs (Day 0) compared to cardiac differentiation groups (Day 3, 6, 9 and 12) (|log2FC| ≥ 1.2, *P* < 0.05, n = 3).

**(B)** KEGG pathway analysis of significantly down-regulated miRNAs in 12-day cardiac-differentiated hESCs.

**(C)** GO analysis of significantly down-regulated miRNAs in 12-day cardiac-differentiated hESCs. The previously reported signaling pathways related to cardiac development have been highlighted in red.


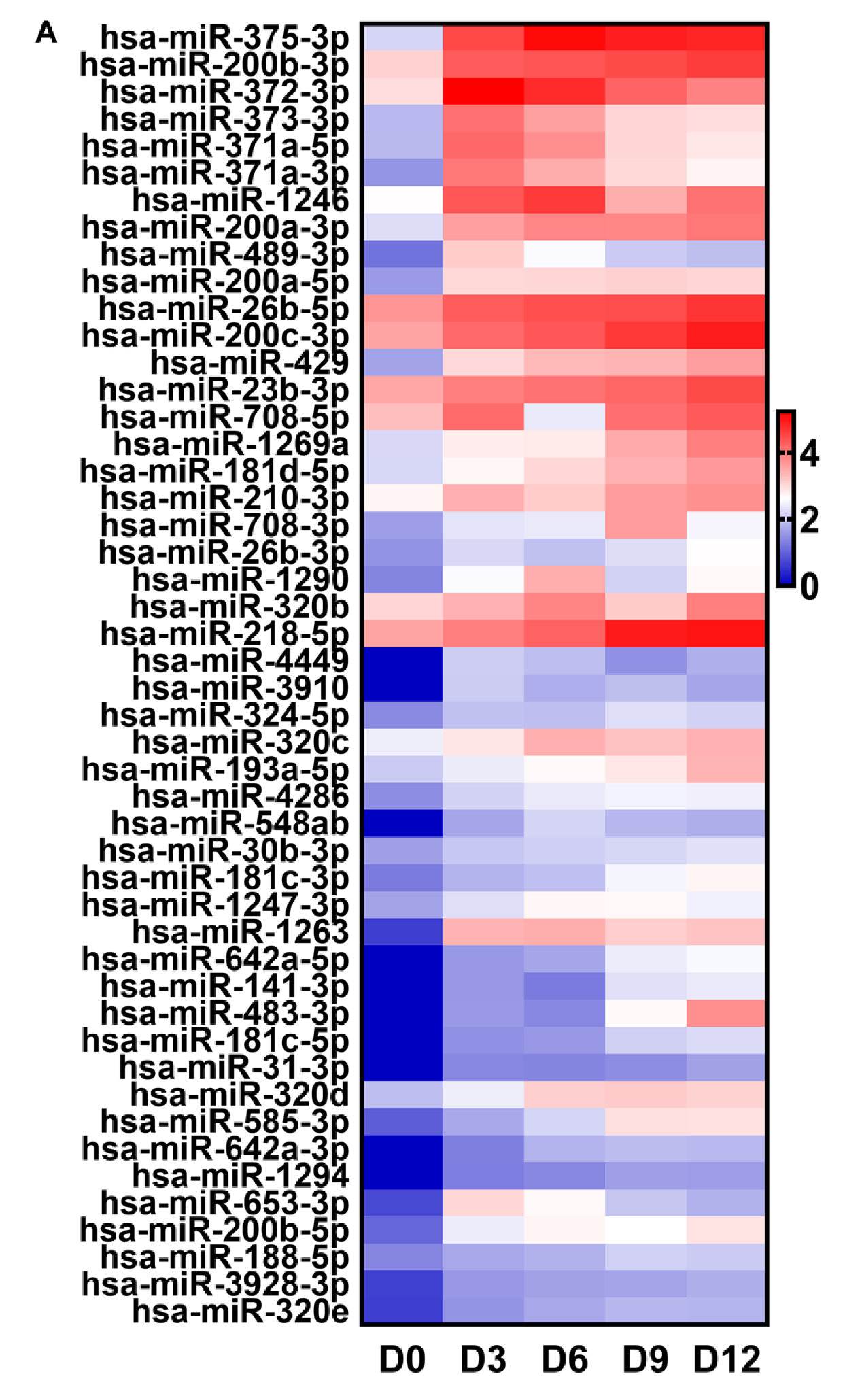


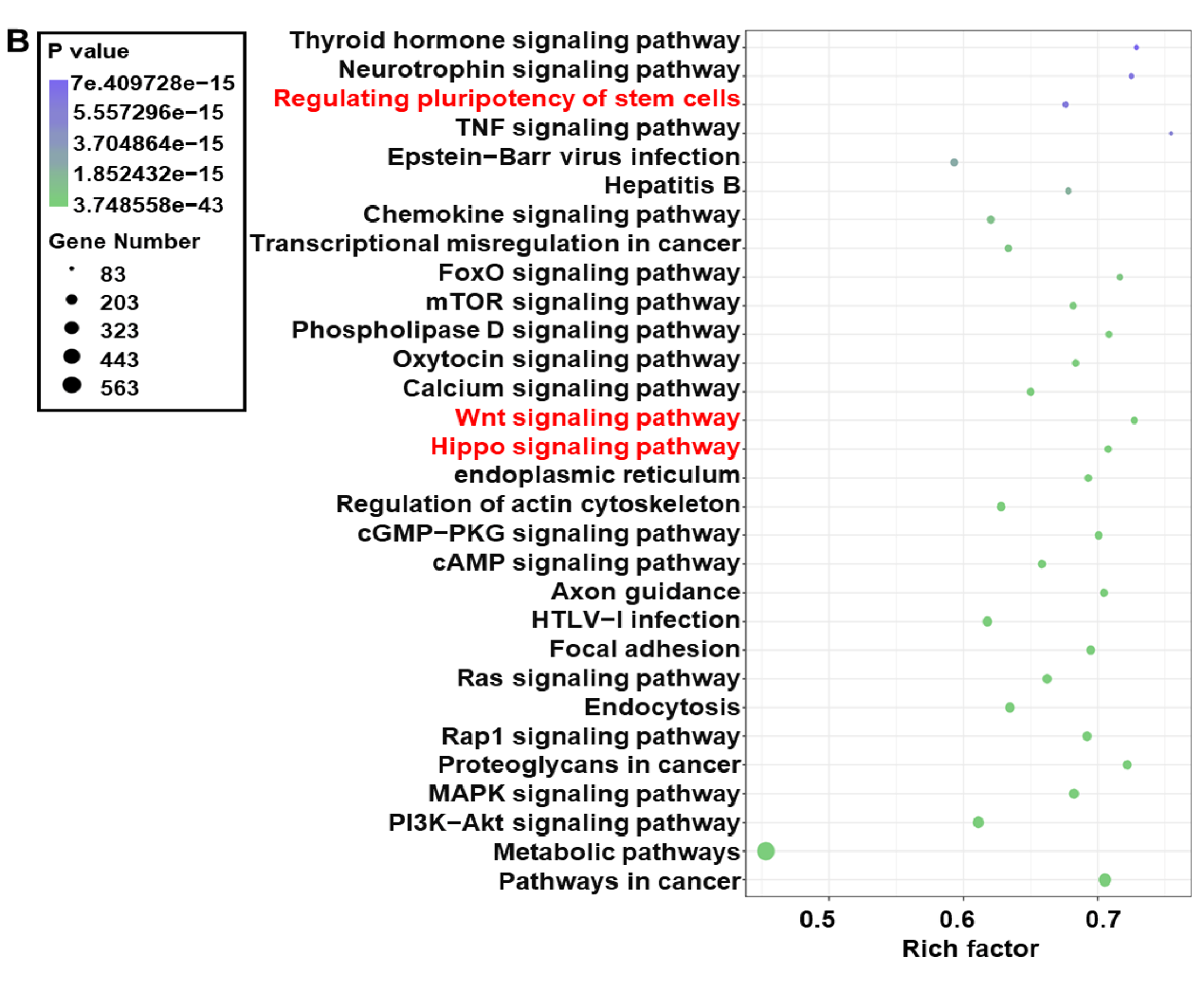


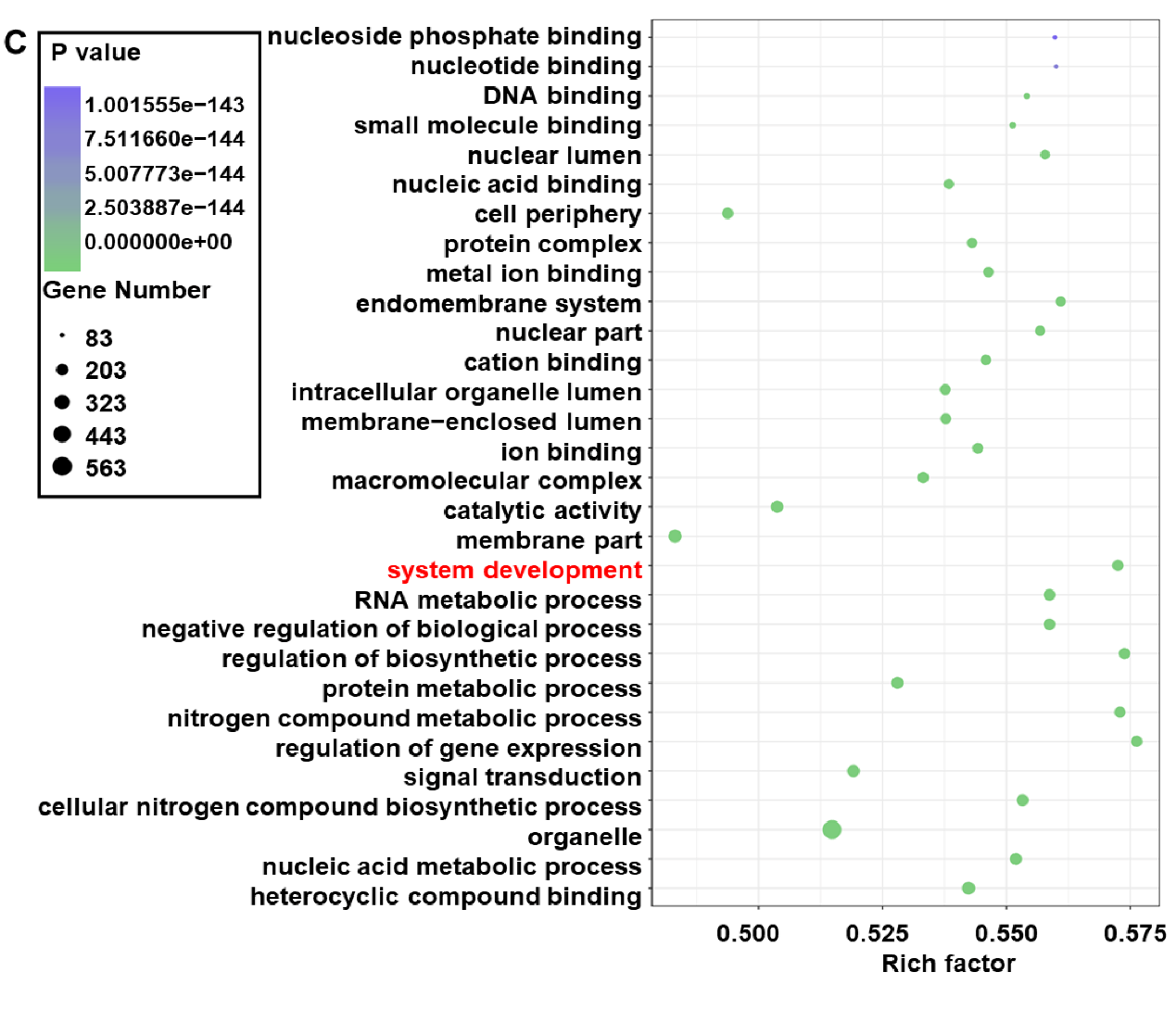


**Figure S3 RNA sequencing with KEGG and GO analysis of miRNAs up-regulated during 12-day hESCs-derived cardiac differentiation.
(A)** Heatmap illustrated the expression levels of 48 miRNAs upregulated in hESCs (Day 0) compared to cardiac differentiation groups (Day 3, 6, 9 and 12) (|log2FC| ≥ 1.2, *P* < 0.05, n = 3).

**(B)** KEGG pathway analysis of significantly up-regulated miRNAs in 12-day cardiac-differentiated hESCs.
**(C)** GO analysis of significantly up-regulated miRNAs in 12-day cardiac-differentiated hESCs. The previously reported signaling pathways related to cardiac development have been highlighted in red.

**
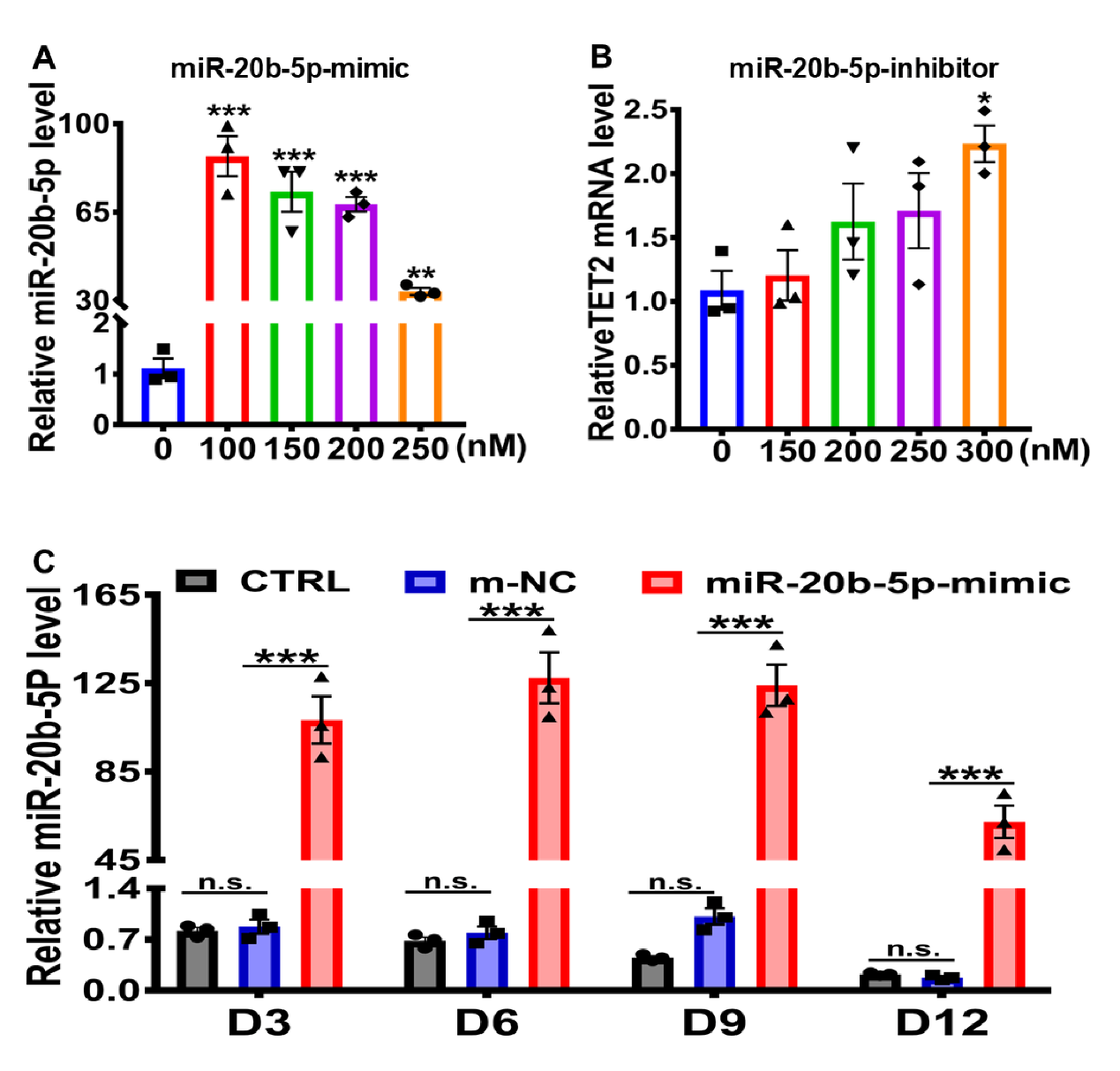
**

**Figure S4 Identification of the transfection efficiency and effect of miR-20b-5p mimic and inhibitor** **during 12-day cardiac differentiation of hESCs.**

**(A)** qRT-PCR analysis of miR-20b-5p level in hESCs transfected with a serial of dosages (0, 100, 150, 200 or 250 nM) of miR-20b-5p mimic for 72 hours (n = 3).

**(B)** qRT-PCR analysis of relative *TET2* mRNA level in hESCs transfected with a serial of dosages (0, 150, 200, 250 or 300 nM) of miR-20b-5p inhibitor for 72 hours (n = 3).

**(C)** qRT-PCR analysis of miR-20b-5p level during 12-day cardiac differentiation of hESCs with transfection of 100 nM mimic NC (m-NC) or miR-20b-5p-mimic (n = 3). Transfection reagent was changed every two days throughout the 12-day cardiac differentiation. Quantitative data were presented as mean ± SEM. Statistical significance was analyzed via a one-way ANOVA followed by Bonferroni multiple comparisons test and represented as **P* < 0.05, ***P* < 0.01 and ****P* < 0.001, while n.s. indicated non-significance.


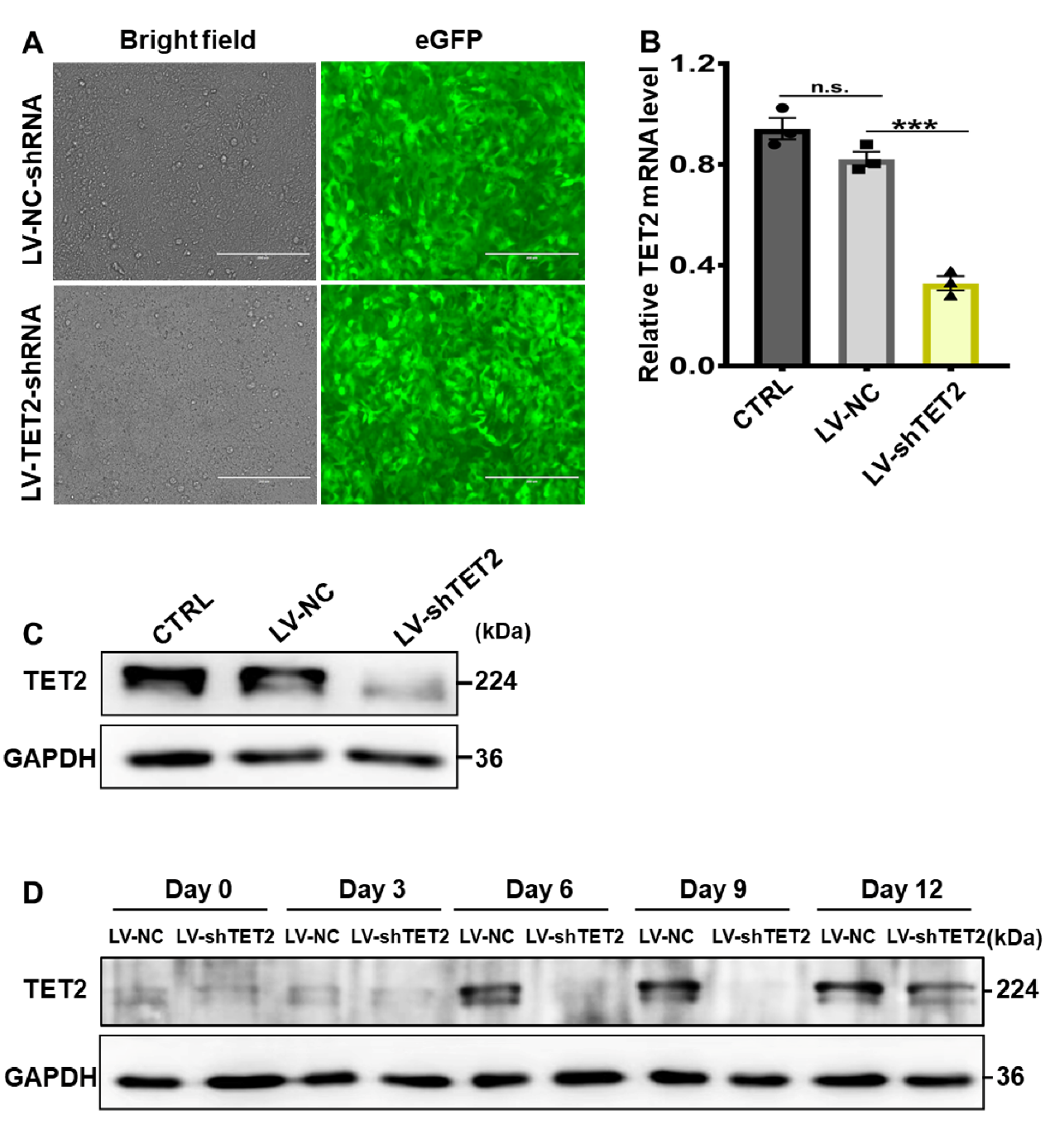
**Figure S5** **Verification of the TET2 knockdown efficiency during 12-day cardiac differentiation of hESCs.**

**(A)** Fluorescence images of hESCs infected with either lentivirus (LV)-NC or LV-TET2 shRNA with enhanced green fluorescent protein (eGFP). (Scale bar = 200 μm.)

**(B)** qRT-PCR analysis of the efficiency of *TET2* knockdown in hESCs infected with LV-NC or LV-TET2 shRNA (n = 3).

**(C)** Western blot analysis of the efficiency of TET2 knockdown in hESCs infected with LV-NC or LV-TET2 shRNA.

**(D)** Western blot analysis of the efficiency of TET2 knockdown during 12-day cardiac differentiation of hESCs with infection of LV-NC or LV-TET2 shRNA.

Quantitative data were presented as mean ± SEM. Statistical significance was analyzed via a one-way ANOVA followed by Bonferroni multiple comparisons test and represented as ****P* < 0.001, while n.s. indicated non-significance.


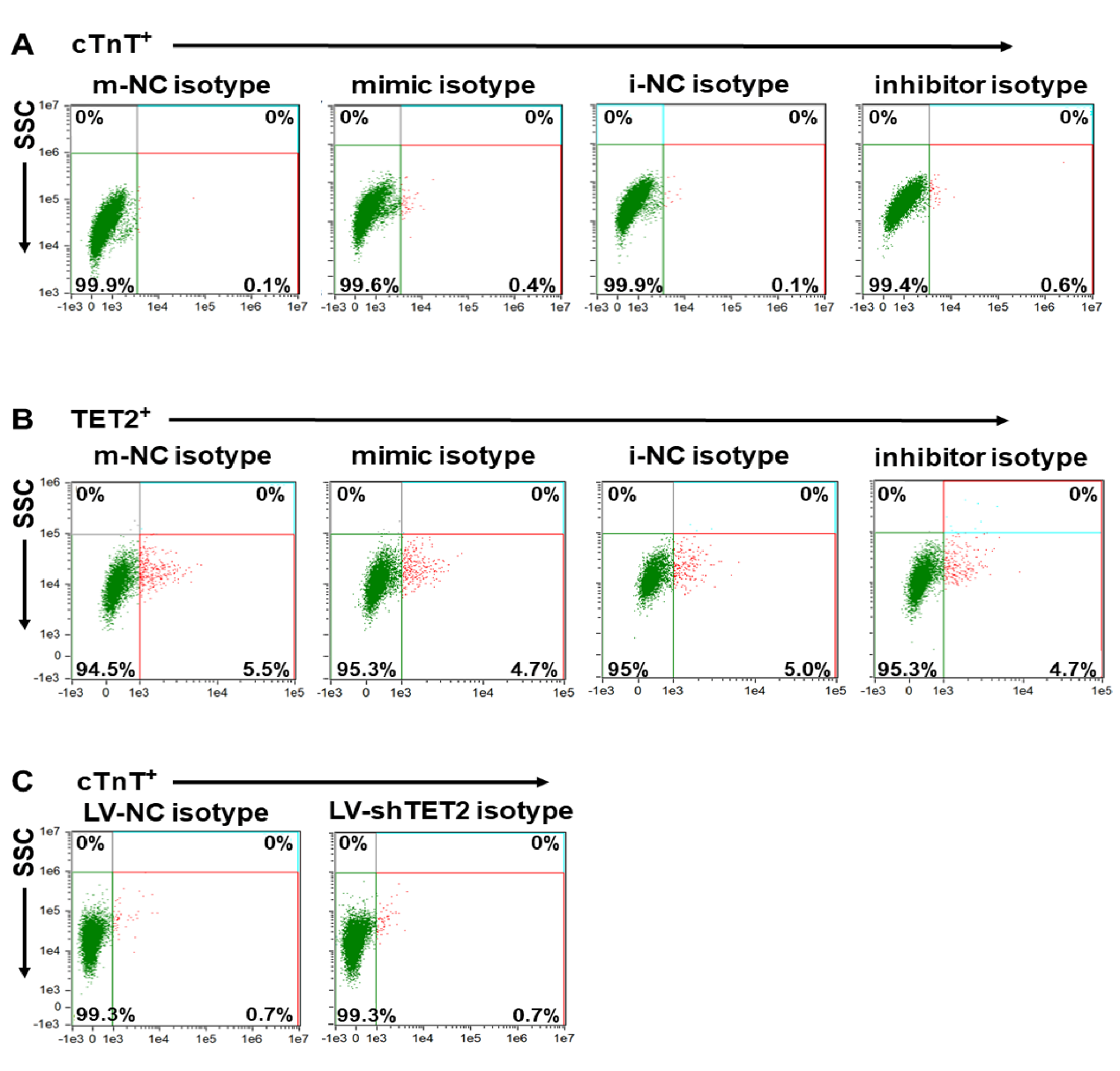


**Figure S6 Establishment of cell population gating based on isotype controls in flow cytometry analysis.**

**(A)** Flow cytometry analysis of cTnT-positive cells in 12-day cardiac-differentiated hESCs from isotype group with transfection of mimic NC (m-NC), miR-20b-5p-mimic, inhibitor NC (i-NC) or miR-20b-5p-inhibitor.

**(B)** Flow cytometry analysis of TET2-positive cells in 12-day cardiac-differentiated hESCs from isotype group with transfection of m-NC, miR-20b-5p-mimic, i-NC or miR-20b-5p-inhibitor.

**(C)** Flow cytometry analysis of cTnT-positive cells in 12-day cardiac-differentiated hESCs from isotype group with transfection of LV-NC or LV-shTET2 shRNA.

**Supplemental tables**

**Table S1 The expression levels of significantly down-regulated miRNAs relative to cardiovascular disease during 12-day cardiac differentiation of hESCs.**

| **Screened miRNAs** | **Day 3** vs Day 0 | | **Day 6 vs Day 0** | | **Day 9** vs Day 0 | | **Day 12 vs Day 0** | |
| --- | --- | --- | --- | --- | --- | --- | --- | --- |
|  | **log2 FC** | ***P* Value** | **log2 FC** | ***P* Value** | **log2 FC** | ***P* Value** | **log2 FC** | ***P* Value** |
| **hsa-miR-129-5p** | **-3.336964167** | **9.83E-194** | **-1.242536087** | **3.32E-15** | **-1.783553492** | **3.98E-36** | **-1.893802885** | **1.20E-46** |
| **hsa-miR-335-3p** | **-3.635974422** | **4.10E-87** | **-3.787675138** | **1.77E-97** | **-3.770725643** | **5.13E-42** | **-3.904148309** | **5.51E-58** |
| **hsa-miR-222-3p** | **-1.685675196** | **1.16E-64** | **-2.612809095** | **6.50E-293** | **-3.210846071** | **7.92E-302** | **-2.663964022** | **3.31E-244** |
| **hsa-miR-486-5p** | **-1.863722762** | **1.99E-52** | **-2.631235549** | **9.35E-95** | **-1.665604282** | **0.017574861** | **-2.508129166** | **1.30E-12** |
| **hsa-miR-92b-3p** | **-1.77697923** | **6.24E-46** | **-1.781244101** | **4.86E-49** | **-3.210688204** | **6.70E-111** | **-2.56095868** | **1.11E-39** |
| **hsa-miR-155-5p** | **-1.491399523** | **1.02E-39** | **-2.262550309** | **5.22E-86** | **-2.050434813** | **8.06E-68** | **-2.150757935** | **7.12E-52** |
| **hsa-miR-4454** | **-3.546676069** | **2.95E-34** | **-10.52390055** | **2.03E-18** | **-4.589767016** | **1.62E-05** | **-4.869606731** | **7.69E-53** |
| **hsa-miR-221-5p** | **-1.975871626** | **1.16E-22** | **-3.62219235** | **3.35E-67** | **-4.345848799** | **1.50E-75** | **-4.273928481** | **1.07E-78** |
| **hsa-miR-378a-5p** | **-1.366683931** | **5.15E-16** | **-1.27631251** | **6.01E-15** | **-1.782444848** | **4.42E-11** | **-1.880137393** | **4.45E-19** |
| **hsa-miR-199b-5p** | **-1.514832634** | **1.80E-15** | **-1.586304949** | **1.52E-20** | **-1.664059737** | **9.50E-14** | **-1.536557247** | **5.24E-11** |
| **hsa-let-7i-5p** | **-1.593129789** | **4.77E-12** | **-1.788794439** | **5.33E-15** | **-2.45438296** | **9.51E-17** | **-1.846436887** | **5.72E-06** |
| **hsa-miR-206** | **-10.48188027** | **8.42E-11** | **-10.73698485** | **4.39E-11** | **-4.231081802** | **0.011389543** | **-4.629602708** | **6.22E-13** |
| **hsa-miR-663a** | **-7.853912825** | **6.52E-10** | **-8.100365196** | **1.57E-10** | **-3.24888552** | **0.011337538** | **-1.512690344** | **0.003586362** |
| **hsa-miR-20b-5p** | **-1.233765555** | **1.11E-08** | **-2.281356967** | **2.32E-61** | **-3.794545601** | **4.73E-114** | **-2.505988223** | **6.18E-16** |
| **hsa-miR-124-5p** | **-4.587451493** | **1.09E-56** | **-4.01569969** | **2.38E-59** | **-11.79695483** | **1.33E-22** | **-5.215230011** | **0.000191721** |

**Table S2 The expression levels of significantly up-regulated miRNAs associated with cardiovascular disease during 12-day cardiac differentiation of hESCs.**

| **Screened miRNAs** | **Day 3** vs Day 0 | | **Day 6 vs Day 0** | | **Day 9** vs Day 0 | | **Day 12 vs Day 0** | |
| --- | --- | --- | --- | --- | --- | --- | --- | --- |
|  | **log2 FC** | ***P* Value** | **log2 FC** | ***P* Value** | **log2 FC** | ***P* Value** | **log2 FC** | ***P* Value** |
| **hsa-miR-375-3p** | **7.810965791** | **0** | **9.625680364** | **0** | **8.610056497** | **0** | **7.968065141** | **2.72E-247** |
| **hsa-miR-200b-3p** | **4.143625325** | **0** | **4.127605548** | **0** | **4.080051313** | **8.97E-83** | **4.16000776** | **0** |
| **hsa-miR-372-3p** | **7.670156461** | **0** | **5.955013716** | **0** | **3.730952902** | **1.30E-100** | **2.195367724** | **2.21E-33** |
| **hsa-miR-371a-5p** | **7.697600198** | **0** | **6.123527393** | **3.68E-237** | **3.393480898** | **3.12E-40** | **3.824241891** | **6.15E-50** |
| **hsa-miR-1246** | **7.697600198** | **0** | **6.518376529** | **0** | **2.509676648** | **1.72E-06** | **4.895527951** | **3.34E-292** |
| **hsa-miR-200a-3p** | **4.55672412** | **6.34E-240** | **5.147536069** | **1.13E-276** | **4.775357232** | **3.05E-101** | **3.971587378** | **9.72E-75** |
| **hsa-miR-200a-5p** | **4.923118887** | **2.84E-158** | **4.769222679** | **3.38E-148** | **4.575605826** | **7.22E-60** | **2.292852352** | **3.75E-69** |
| **hsa-miR-26b-5p** | **2.040143454** | **1.19E-105** | **2.225089561** | **2.10E-142** | **2.061332349** | **3.47E-46** | **3.631663405** | **0** |
| **hsa-miR-200c-3p** | **2.060197799** | **1.65E-90** | **2.450017089** | **6.49E-220** | **3.075484245** | **2.23E-74** | **5.583311049** | **4.18E-155** |
| **hsa-miR-429** | **4.617719076** | **4.89E-79** | **5.502927692** | **2.06E-139** | **5.222370667** | **1.24E-68** | **2.25886003** | **1.41E-70** |
| **hsa-miR-23b-3p** | **1.52593165** | **6.88E-70** | **1.67909972** | **2.34E-71** | **1.799379255** | **1.86E-32** | **1.349737735** | **5.14E-15** |
| **hsa-miR-708-5p** | **2.940207383** | **4.21E-52** | **1.500814935** | **1.13E-15** | **2.298149963** | **4.92E-25** | **2.4893056** | **2.10E-33** |
| **hsa-miR-181d-5p** | **1.809883936** | **1.31E-38** | **2.596795135** | **3.14E-109** | **3.532663955** | **1.14E-51** | **3.951991301** | **1.68E-85** |
| **hsa-miR-210-3p** | **2.486873751** | **3.69E-35** | **1.279445422** | **4.70E-20** | **2.62853188** | **1.25E-49** | **2.494456099** | **6.63E-84** |
| **hsa-miR-708-3p** | **2.522899372** | **4.19E-32** | **2.481989942** | **2.13E-32** | **2.552765916** | **8.82E-25** | **2.099131486** | **1.81E-24** |
| **hsa-miR-26b-3p** | **2.509626516** | **4.94E-27** | **1.432641533** | **7.56E-08** | **2.12234342** | **5.61E-13** | **2.911802926** | **3.44E-31** |
| **hsa-miR-320b** | **1.310030447** | **1.37E-22** | **2.567131679** | **1.22E-86** | **1.879656359** | **1.32E-27** | **2.020454432** | **1.71E-30** |
| **hsa-miR-218-5p** | **1.388080096** | **1.20E-17** | **2.105682346** | **7.87E-277** | **4.238665229** | **2.72E-82** | **3.95627708** | **5.87E-192** |
| **hsa-miR-324-5p** | **2.056363329** | **1.36E-14** | **1.685573187** | **1.33E-08** | **2.632216031** | **7.03E-12** | **1.581099553** | **7.92E-09** |
| **hsa-miR-193a-5p** | **1.27299331** | **2.19E-11** | **1.916817707** | **2.43E-27** | **2.149955493** | **2.02E-24** | **3.459253466** | **5.90E-54** |
| **hsa-miR-30b-3p** | **1.506670324** | **1.63E-10** | **1.565190119** | **2.77E-12** | **1.460392195** | **2.80E-10** | **1.325940761** | **4.01E-11** |
| **hsa-miR-1247-3p** | **2.184256879** | **2.00E-10** | **3.373151233** | **3.16E-50** | **2.902100959** | **6.51E-15** | **1.72126204** | **2.17E-07** |
| **hsa-miR-141-3p** | **7.660943915** | **1.75E-09** | **8.556971692** | **9.99E-12** | **9.642440685** | **3.82E-13** | **9.43486435** | **2.39E-15** |
| **hsa-miR-483-3p** | **7.685364033** | **3.64E-09** | **6.81178104** | **2.61E-06** | **10.77572176** | **1.69E-18** | **13.97002189** | **1.13E-30** |
| **hsa-miR-181c-5p** | **7.428051284** | **3.42E-08** | **7.438452878** | **5.95E-09** | **9.054746** | **1.69E-13** | **8.97736065** | **7.94E-14** |
| **hsa-miR-320d** | **1.864274** | **3.28E-07** | **3.835864268** | **5.19E-32** | **3.743746216** | **7.92E-05** | **2.935956137** | **1.59E-16** |
| **hsa-miR-188-5p** | **1.290542567** | **0.000217456** | **1.385087653** | **4.93E-05** | **2.232714592** | **9.86E-07** | **1.465877236** | **2.81E-06** |

**Table S3 The expression levels of eight candidate miRNAs in plasma samples from ASD patients.**

| **Screened miRNA** | **FC(ASD/Control)** |  | **Log2FC(ASD/Control)** | **Prob Value** |  |
| --- | --- | --- | --- | --- | --- |
|  |  |  |  |  |  |
| **hsa-miR-20b-5p** | **1.543946129** |  | **0.626622415** | **0.953016849** | |
| **hsa-miR-3173-5p** | **2.598252054** |  | **1.377541392** | **0.925447667** | |
| **hsa-miR-222-5p** | **3.561055532** |  | **1.832304933** | **0.905763347** | |
| **hsa-miR-335-3p** | **2.093557197** |  | **1.065956334** | **0.93344115** | |
| **hsa-miR-378a-5p** | **1.880641605** |  | **0.91122494** | **0.94163461** | |
| **hsa-miR-4746-5p** | **2.05500075** |  | **1.03913892** | **0.903803051** | |
| **hsa-miR-486-5p** | **1.805191562** |  | **0.85215194** | **0.979095278** | |
| **hsa-miR-92b-3p** | **1.718499808** |  | **0.78114969** | **0.999003358** | |

**Table S4 The expression levels of eight candidate miRNAs during 12-day cardiac-differentiation of hESCs.**

| **Screened miRNAs** | **Day 3** vs Day 0 | | **Day 6 vs Day 0** | | **Day 9** vs Day 0 | | **Day 12 vs Day 0** | |
| --- | --- | --- | --- | --- | --- | --- | --- | --- |
|  | **log2 FC** | ***P* Value** | **log2 FC** | ***P* Value** | **log2 FC** | ***P* Value** | **log2 FC** | ***P* Value** |
| **hsa-miR-20b-5p** | **-1.233765555** | **1.11E-08** | **-2.28135697** | **2.32E-61** | **-3.794545601** | **4.73E-114** | **-2.50598822** | **6.70E-111** |
| **hsa-miR-3173-5p** | **-1.497904559** | **-1.497904559** | **-2.63123555** | **1.09E-10** | **-1.692809066** | **6.90E-05** | **-1.21554267** | **1.85E-05** |
| **hsa-miR-222-5p** | **-1.685675196** | **1.16E-64** | **-1.94766603** | **2.12E-17** | **-3.186564798** | **2.84E-07** | **-2.66396402** | **2.42E-18** |
| **hsa-miR-335-3p** | **-2.150487416** | **7.40E-12** | **-3.78767514** | **1.77E-97** | **-1.163035738** | **5.13E-42** | **-3.90414831** | **5.51E-58** |
| **hsa-miR-378a-5p** | **-1.232493641** | **9.69E-44** | **-1.27631251** | **6.01E-15** | **-1.184122825** | **1.19E-11** | **-2.06499978** | **4.45E-19** |
| **hsa-miR-4746-5p** | **-1.327026225** | **9.51E-08** | **-1.49524613** | **1.15E-10** | **-1.230819865** | **2.57E-06** | **-2.25767068** | **8.70E-24** |
| **hsa-miR-486-5p** | **-1.863722762** | **1.99E-52** | **-2.63123555** | **9.35E-95** | **-1.665604282** | **0.017574861** | **-2.50812917** | **1.30E-12** |
| **hsa-miR-92b-3p** | **-1.77697923** | **6.24E-46** | **-1.7812441** | **4.86E-49** | **-3.210688204** | **6.70E-111** | **-3.04978948** | **1.11E-39** |

**Table S5 TargetScan predicted the potential of screened miRNAs targeting TET2.**

| **Screened miRNAs** | **Predicted target region in TET2** | **Site type** | **Context score** | **Context score percentile** | **Weighted context score** | **Conserved branch length** | **P_CT_** |
| --- | --- | --- | --- | --- | --- | --- | --- |
| **hsa-miR-20b-5p** | **3232-3238** | **7mer-m8** | **-0.08** | **86** | **-0.08** | **4.072** | **0.72** |
| **hsa-miR-335-3p** | **790-796** | **7mer-m8** | **-0.02** | **61** | **-0.02** | **0.031** | **N/A** |
| **hsa-miR-486-5p** | **2874-2880** | **7mer-1A** | **-0.01** | **56** | **-0.01** | **1.403** | **N/A** |
| **hsa-miR-222-5p** | **2799-280** | **7mer-1A** | **-0.02** | **32** | **-0.02** | **0** | **N/A** |
| **hsa-miR-3173-5p** | **3838-3849** | **non-canonical** | **N/A** | **N/A** | **N/A** | **0** | **N/A** |
| **hsa-miR-4746-5p** | **-** | **-** | **-** | **-** | **-** | **-** | **-** |
| **hsa-miR-378a-5p** | **-** | **-** | **-** | **-** | **-** | **-** | **-** |
| **hsa-miR-92b-3p** | **-** | **-** | **-** | **-** | **-** | **-** | **-** |

**Table S6 The mature sequence of miR-20b-5p in human or mouse species.**

| **Species** | **Mature sequence** |
| --- | --- |
| **hsa-miR-20b-5p** | **CAAAGUGCUCAUAGUGCAGGUAG** |
| **mmu-miR-20b-5p** | **CAAAGUGCUCAUAGUGCAGGUAG** |

**Table S7 Patient Characteristics.**

| **Ages (years)** | **Sex** | **Atrial septal defect (ASD)** |
| --- | --- | --- |
| **8** | **Male** | **Yes** |
| **3.6** | **Female** | **Yes** |
| **3.6** | **Female** | **Yes** |
| **5** | **Female** | **Yes** |
| **5.5** | **Female** | **Yes** |
| **4** | **Female** | **Yes** |
| **2.7** | **Female** | **Yes** |
| **3.6** | **Male** | **Yes** |
| **3.6** | **Female** | **Yes** |
| **2.6** | **Female** | **Yes** |
| **4** | **Male** | **No** |
| **5** | **Male** | **No** |
| **3.3** | **Female** | **No** |
| **2** | **Female** | **No** |
| **3** | **Female** | **No** |
| **3** | **Female** | **No** |
| **4** | **Female** | **No** |
| **2** | **Male** | **No** |
| **3** | **Male** | **No** |

**Table S8 The target sequences of shRNA.**

| **Name** | **Target Sequences** |
| --- | --- |
| **Negative control shRNA** | **CCTAAGGTTAAGTCGCCCTCG** |
| **TET2 shRNA** | **TTTCACGCCAAGTCGTTATTT** |

**Table S9 The primers of real‐time PCR used in this study.**

| **Genes** | **Forward Primer (5’-3’)** | **Reverse Primer (5’-3’)** |  |
| --- | --- | --- | --- |
| ***hsa-TET2*** | **GATAGAACCAACCATGTTGAGGG** | **TGGAGCTTTGTAGCCAGAGGT** | |
| ***hsa-NKX2.5*** | **CTATCCGGGTTACGGCGG** | **TGAACCGCATTCAAGTCCCC** | |
| ***hsa-GATA4*** | **CGACACCCCAATCTCGATATGTT** | **ACAGATAGTGACCCGTCCCA** | |
| ***hsa-TBX5*** | **TACCACCACACCCATCAAC** | **ACACCAAGACAGGGACAGAC** | |
| ***hsa-MYH6*** | **CAAGAGCCGTGACATTGGTG** | **AGGTTGGCAAGAGTGAGGTT** | |
| ***hsa-cTnT*** | **AGACGCCTCCAGGATCTGT** | **TCTTCAACAGCTGCTTCTTCC** | |
| ***hsa-GAPDH*** | **ACCACAGTCCATGCCATCAC** | **CATGCCAGTGAGCTTCCCGT** | |
| ***mmu-TET2*** | **AAGCTGATGGAAAATGCAAGC** | **GCTGAAGGTGCCTCTGGAGT** | |
| ***mmu-GAPDH*** | **TGGTGAAGGTCGGTGTGAAC** | **GCTCCTGGAAGATGGTGATGG** | |
